# Investigating the impact of poly(beta amino) ester-mediated *FOXJ1* mRNA delivery on differentiation of primary human bronchial epithelial cells

**DOI:** 10.1242/bio.062419

**Published:** 2026-06-12

**Authors:** Rafaela Konstantinidi, Mira Subramanian, Laura Yates, Clare M. Lloyd, Sejal Saglani, Asha K. Patel

**Affiliations:** ^1^ National Heart and Lung Institute, Imperial College London, London W12 0NN, UK; ^2^ Royal Brompton Hospital, Guy's and St Thomas’ NHS Foundation Trust, London SW3 6NP, UK; ^3^ Centre for Paediatrics and Child Health, Imperial College London, London SW7 2AZ, UK

**Keywords:** mRNA, Transcription factor, FOXJ1, Primary human bronchial epithelial cells, Air-liquid interface culture, Differentiation

## Abstract

Primary human basal bronchial epithelial cells (HBECs) are an important population of progenitor cells capable of self-renewal and differentiation to maintain airway homeostasis. In air-liquid interface (ALI) culture, HBECs undergo mucociliary differentiation, providing a robust physiologic model to evaluate novel therapeutics such as *in vitro*-transcribed messenger RNA (IVT-mRNA). However, the impact of IVT-mRNA delivery on the differentiation potential of basal HBECs remains poorly characterised. Poly(beta amino) ester (PBAE) nanoparticles have demonstrated effective airway delivery of IVT-mRNA in various preclinical studies. Here, we aimed to understand the impact of PBAE-mediated mRNA transfection on basal HBEC differentiation at the ALI. We investigated IVT-mRNA encoding the ciliogenesis transcription factor Forkhead box J1 (FOXJ1) as a model tool for transient overexpression in primary basal HBECs and characterised its subsequent impact on epithelial integrity and differentiation at the ALI. PBAE-mediated delivery of *FOXJ1* mRNA to submerged primary HBECs resulted in approximately 50% FOXJ1-positive cells and transient upregulation of key ciliogenesis-related genes, including *DNALI1* and *RSPH9*. Following 28 days of differentiation at the ALI, *FOXJ1*- or reporter mRNA-transfected cultures displayed normal epithelial morphology, with tight junction and differentiation markers, proportions of secretory and ciliated cells, and ciliary ultrastructure comparable to those of non-transfected controls. These data indicate that PBAE-mediated IVT-mRNA delivery can transiently increase encoded protein expression in basal primary HBECs without impeding mucociliary differentiation.

## Teaser

**Summary:** Polymer nanoparticle-mediated delivery of mRNA to primary human bronchial basal cells facilitates transient protein production without compromising epithelial barrier integrity or mucociliary differentiation at the air-liquid interface.

## INTRODUCTION

The airway epithelium forms a critical barrier between the external environment and the internal respiratory system, providing the first line of defence against inhaled pathogens, pollutants, toxins, and allergens (Tam et al., 2011). This pseudostratified tissue consists primarily of basal, secretory, and ciliated cells, which together maintain airway homeostasis (Ross et al., 2007; Vareille et al., 2011; Hewitt and Lloyd, 2021). Secretory cells, including goblet and club cells, contribute to the biochemical barrier through secretion of mucus and antimicrobial peptides that entrap pathogens and facilitate their clearance from the respiratory tract (Boers et al., 2012; Ma et al., 2018). Multiciliated cells promote mucociliary clearance by coordinated beating of their motile apical cilia, thereby removing mucus and entrapped particles from the airway surface (Rawlins and Hogan, 2008; Bustamante-Marin and Ostrowski, 2017). The structural and functional integrity of the epithelium is further maintained by intracellular junctional complexes, including tight junctions, adherens junctions, and desmosomes, which regulate paracellular permeability and limit penetration of harmful agents (Roche et al., 1993; Brune et al., 2015). Bronchial epithelial basal cells constitute the principal progenitor population of the conducting airways. They sustain epithelial turnover during homeostasis and activate after injury to proliferate, self-renew and differentiate to repair and restore mucociliary function (Rock et al., 2009, 2010). Given their central role in epithelial repair and differentiation, primary basal cells provide a relevant system in which to assess how emerging nucleic acid-based therapies can impact airway basal cell function.

Airway delivery of emerging therapeutics such as mRNA is an attractive strategy to deliver encoded proteins for various applications, including infectious disease, oncology and tissue repair for localised therapy and reduced systemic effects (Huang et al., 2025; Liang et al., 2025; Zhen et al., 2026). Among non-viral delivery systems, poly(beta amino) esters (PBAEs) are promising for pulmonary mRNA delivery (Patel et al., 2019; Blanchard et al., 2021; Vanover et al., 2022; Jiang et al., 2024). Preclinical studies have shown that nebulised PBAE-mRNA formulations can support lung transgene expression and therapeutic activity. Patel et al. (2019) demonstrated that nebulised hyperbranched PBAE-mRNA polyplexes drive robust reporter protein expression in the mouse lung epithelium, with repeat dosing achieved without overt toxicity. Blanchard et al. (2021) later showed that nebulised PBAE-formulated *Cas13a* mRNA had antiviral activity against influenza in rodents, while Vanover et al. reported that nebulised mRNA encoding neutralising antibodies protected hamsters from SARS-CoV-2 infection.

Despite these encouraging *in vivo* findings, the impact of transient PBAE-mediated mRNA transfection on the differentiation capacity of primary human basal bronchial epithelial cells (HBECs), in ALI culture, remains poorly characterised. This is a key consideration when using mRNA to modulate gene expression in progenitor populations, where even modest vector-associated perturbations could alter epithelial phenotype and function.

To address this, we used GFP reporter mRNA to establish PBAE-mediated mRNA delivery in primary basal HBECs and to determine whether transient reporter mRNA transfection impacts epithelial integrity and subsequent differentiation under ALI culture conditions. We then evaluated *in vitro*-transcribed messenger RNA (IVT-mRNA) encoding *FOXJ1* as a biologically relevant endogenous transcription factor payload. FOXJ1 is a master regulator of motile ciliogenesis (Pelletier et al., 1998; You et al., 2004; Yu et al., 2008) and controls the expression of genes required for ciliary assembly and function, including axonemal structural components, intraflagellar transport machinery, and dynein motor proteins (Choksi et al., 2014; Mukherjee et al., 2019). Dysregulated FOXJ1 expression has been linked to defective ciliogenesis and impaired ciliary function in conditions such as primary ciliary dyskinesia (PCD) and bronchiectasis (Peng et al., 2018; Wallmeier et al., 2019; Shapiro et al., 2021; Zou et al., 2023). Manipulation of FOXJ1 *in vitro* has provided important insights into its role in ciliogenesis. Plasmid-mediated FOXJ1 overexpression in human airway epithelial cells induces cilia-associated gene expression, particularly when co-expressed with the transcriptional co-regulator *RFX3* (Didon et al., 2013). Conversely, CRISPR-Cas9-mediated FOXJ1 disruption abolishes motile cilia formation in primary airway epithelial cells (Rapiteanu et al., 2020). Lentiviral overexpression has been shown to restore cilia-related gene expression and protect against cigarette-smoke-extract-induced inhibition of cilia growth (Brekman et al., 2014). However, these approaches rely on DNA-based overexpression or genome editing, which carry risks of genomic integration and prolonged expression.

Here, we investigated the impact of PBAE-mediated delivery of IVT-mRNA on primary basal HBEC differentiation under ALI culture conditions. Using *FOXJ1* mRNA as a representative endogenous transcription factor cargo, we assessed whether IVT-mRNA delivery drives transient *FOXJ1* expression and transcriptional activity, assessed by induction of ciliogenesis-associated genes, while remaining compatible with subsequent HBEC differentiation, including appropriate cell-type composition, cilia formation and tight junction integrity.

## RESULTS

### PBAE-mediated GFP mRNA transfection of primary HBECs before airlift does not impede subsequent differentiation and barrier integrity

**Fig. 1.**
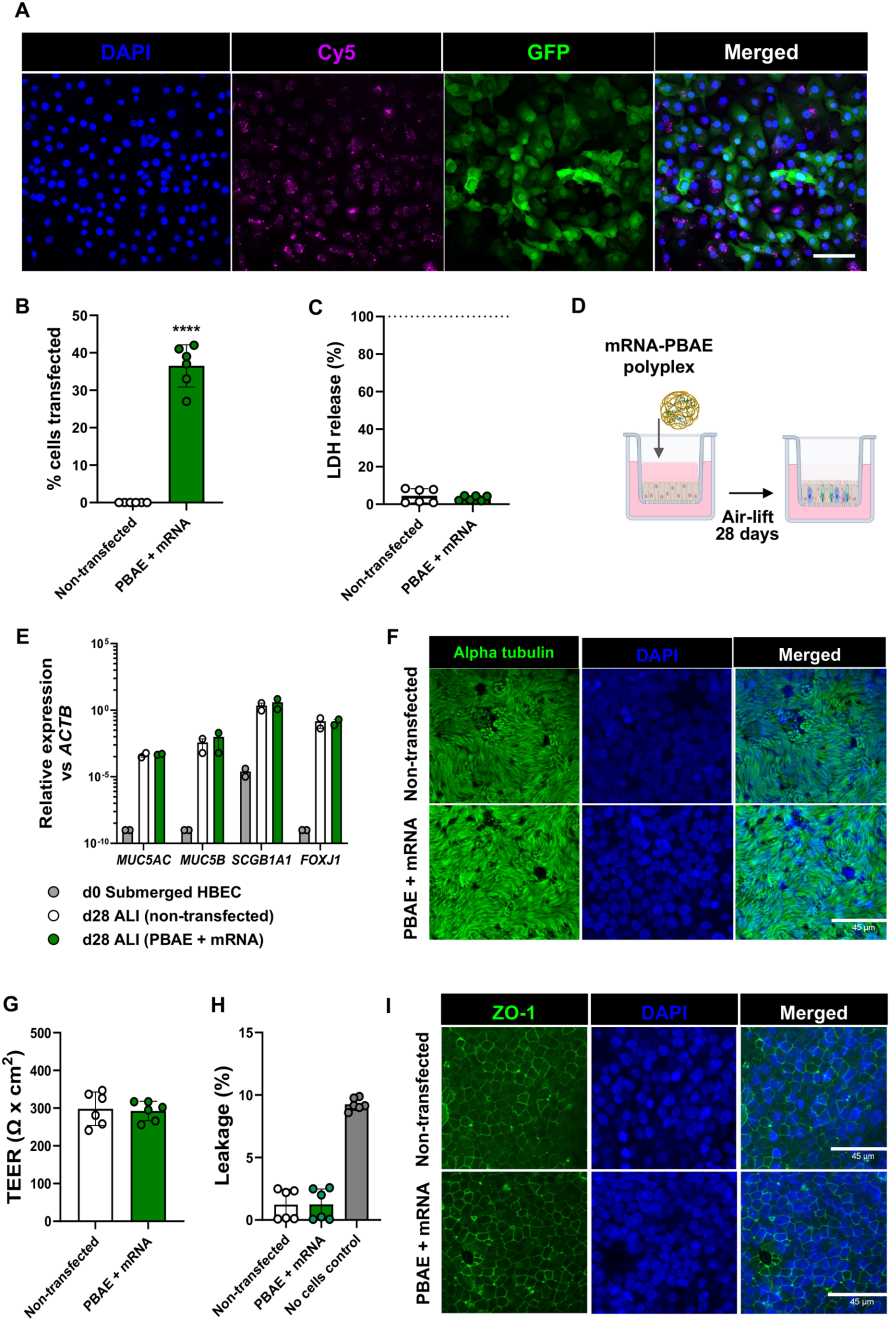
**Epithelial differentiation and barrier integrity remain intact following PBAE-mediated reporter mRNA transfection of primary HBECs.** (A) Representative images of submerged HBECs transfected with Cy5-tagged GFP mRNA complexed with PBAE 24 h post-delivery in eight-well chamber slides, nuclei (DAPI), Cy5 (mRNA uptake) and GFP (mRNA translation). Scale bar: 100 µm. (B) Percentage of Cy5-positive cells indicating percentage of cells transfected at 24 h post-transfection (mean±s.d.). *N*=2 donors, with three replicate wells per donor (*t*-test; ****P<0.0001). (C) Percentage of LDH release at 24 h post-transfection (mean±s.d.) relative to Triton-treated positive control cells (dotted line). *N*=2 donors, with three replicates per donor (*t*-test; *P*=0.47; non-significant). (D) Schematic showing the transfection of submerged HBECs seeded on Transwell inserts and subsequent differentiation at ALI for 28 days. (E) qPCR analysis for secretory cell markers (*MUC5AC*, *MUC5B*, *SCGB1A1*) and a ciliated cell marker (*FOXJ1*) in submerged HBECs and in non-transfected and PBAE-mRNA-transfected HBEC ALI day 28 post-ALI cultures. Results are quantified relative to the housekeeping gene *ACTB*. Data are from *N*=2 independent primary HBEC donors. For each donor, three independent ALI cultures were set up, transfected, and differentiated separately. Lysates from the three independent ALI cultures from each donor were then pooled prior to RNA extraction and qPCR analysis, generating one pooled donor-level sample per donor. Each point represents one pooled lysate derived from three independent ALI cultures from the same donor. Statistical analysis was not performed because only two donor-level samples were available. (F) Representative immunofluorescence staining for acetylated alpha-tubulin in non-transfected and PBAE-mRNA-transfected HBEC day 28 post-ALI cultures. Scale bars: 45 µm. (G) TEER (Ω×cm^2^) was measured across non-transfected HBECs and PBAE-mRNA-transfected HBECs cultured at ALI for 28 days. *N*=2 donors, with three independent ALI cultures established per donor (*t*-test; *P*=0.78; non-significant). (H) Permeability of HBEC ALI cultures to 4 kDa FITC-dextran. No-cell Transwell served as a positive control. *N*=2 donors, with three independent ALI cultures established per donor (*t*-test; *P*=0.98; non-significant). (I) Representative immunofluorescence staining for ZO-1 in non-transfected and PBAE-mRNA-transfected HBEC day 28 post-ALI cultures. Scale bars: 45 µm.

To determine the transfection efficiency of submerged primary HBECs, cells were transfected with Cy5-tagged GFP mRNA complexed with PBAE (Fig. 1A). Efficient cellular uptake of Cy5-mRNA was observed in 37±5.65% of cells (****P<0.0001; Fig. 1B). PBAE-mRNA transfection induced minimal cytotoxicity, as indicated by low lactate dehydrogenase (LDH) release (Fig. 1C).

To assess the impact of reporter mRNA transfection on epithelial differentiation at ALI, primary HBECs were transfected with PBAE-complexed GFP mRNA 24 h after seeding onto Transwell inserts. Following formation of a confluent monolayer, both mRNA-transfected and non-transfected control HBECs were airlifted and cultured at ALI for 28 days (Fig. 1D). Differentiation was evaluated by quantitative PCR (qPCR) analysis of epithelial cell subtype markers, comparing basal submerged cultures at day 0 to differentiated ALI cultures at day 28. Expression of secretory cell markers *MUC5AC*, *MUC5B*, and *SCGB1A1*, as well as the ciliated cell marker *FOXJ1*, was increased in both non-transfected and PBAE-mRNA-transfected cultures after 28 days at ALI, consistent with successful epithelial differentiation (Fig. 1E). Importantly, the expression of these markers was comparable between mRNA-transfected and non-transfected cultures. These findings were supported by immunofluorescence analysis, which indicated that both non-transfected and PBAE-mRNA-transfected HBECs formed a well-differentiated epithelium by day 28 at ALI, as evidenced by the presence of acetylated alpha-tubulin-positive cilia (Fig. 1F). Together, these data suggest that PBAE-mediated reporter mRNA transfection prior to air exposure does not impair differentiation of primary HBECs at ALI.

We next examined whether reporter mRNA transfection affected epithelial barrier integrity. Both groups developed measurable transepithelial electrical resistance (TEER) at day 28, indicating successful epithelial barrier establishment. No significant difference was detected between non-transfected cultures (298.3 Ω×cm^2^±44.4) and PBAE-mRNA-transfected cultures (292.2 Ω×cm^2^±25.8) (*P*=0.78; Fig. 1G). Paracellular permeability was evaluated by measuring the apical-to-basal flux of fluorescein isothiocyanate (FITC)-dextran across differentiated HBEC cultures at day 28 ALI. FITC-dextran permeability was comparable between non-transfected HBECs and PBAE-mRNA-transfected HBECs (Fig. 1H). To visually assess tight junction formation, immunostaining for the ZO-1 protein was performed in day 28 ALI cultures. Both mRNA-transfected and non-transfected cultures showed positive ZO-1 staining, with no observable differences in ZO-1 cellular localisation between groups (Fig. 1I). Collectively, these results demonstrate that reporter mRNA transfection before air exposure does not compromise epithelial barrier integrity in primary HBEC ALI cultures.

### PBAE-mediated *FOXJ1* mRNA transfection of submerged primary HBECs results in FOXJ1 expression and transient induction of ciliogenesis-associated genes

**Fig. 2.**
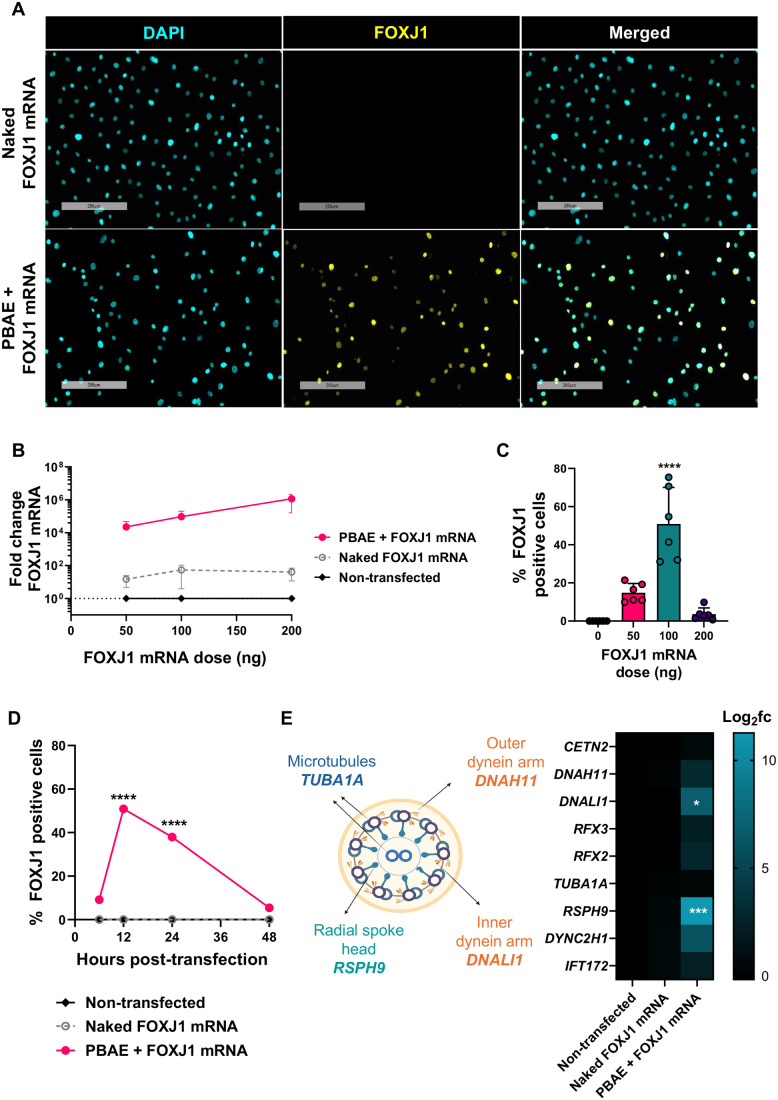
**PBAE-mediated *FOXJ1* mRNA transfection of submerged primary HBECs induces transient expression of ciliogenesis-associated genes.** Submerged primary HBECs in 96-well plates were transfected with increasing doses of *FOXJ1* mRNA, either naked or PBAE-complexed. *N*=3 donors, *n*=3 technical replicates. (A) Representative immunofluorescence images of submerged HBECs at 12 h post-transfection with 100 ng *FOXJ1* mRNA stained for FOXJ1 and DAPI. Scale bars: 200 μm. (B) *FOXJ1* mRNA expression was quantified by qPCR at 12 h post-transfection and displayed as fold change from non-transfected controls (mean±s.d.). (C) Percentage of FOXJ1-positive cells at 12 h post-transfection with varying *FOXJ1* mRNA doses [mean±s.d., *N*=6 donors, one-way analysis of variance (ANOVA) followed by Dunnett's multiple comparisons test, ****P<0.0001]. (D) Time course of average percentage of FOXJ1-positive cells from 6 to 48 h post-transfection (two-way ANOVA followed by Dunnett's multiple comparisons test, ****P<0.0001). (E) Genes selected as representative markers of the *FOXJ1*-regulated ciliogenesis programme, encompassing cilia-related transcription factors (*RFX3*, *RFX2*), structural and motility-associated components (*CETN2*, *DNAH11*, *DNALI1*, *RSPH9*, *TUBA1A*), and intraflagellar transport machinery (*DYNC2H1*, *IFT172*). Created in BioRender by Patel, A. K. (2026). https://BioRender.com/xwmqief. This figure was sublicensed under CC-BY 4.0 terms. qPCR-based expression analysis of selected FOXJ1 downstream genes at 24 h post-transfection with 100 ng *FOXJ1* mRNA (naked or PBAE-complexed) in submerged primary HBECs, displayed as log2 fold change relative to non-transfected controls (*N*=3 donors, *n*=3 technical replicates, two-way ANOVA followed by Dunnett's multiple comparisons test, *P=0.03, ***P=0.0004).

Following successful GFP mRNA transfection of HBECs with PBAE, we then aimed to characterise PBAE nanoparticles for delivery of *FOXJ1* mRNA. PBAE was able to effectively complex *FOXJ1* mRNA at high efficiency as assessed by the RiboGreen assay, with an average particle diameter of 60 nm, a low polydispersity index of 0.2, and a surface charge of 34±18.2 mV (Fig. S1). To assess transfection efficiency under submerged conditions, primary HBECs were transfected with PBAE-formulated *FOXJ1* mRNA at 50, 100 or 200 ng. qPCR demonstrated a dose-dependent increase in *FOXJ1* mRNA levels from 6 to 48 h post-transfection that was not observed with non-complexed ‘naked’ *FOXJ1* mRNA (Fig. 2B; Fig. S2). Immunofluorescence staining confirmed nuclear-localised FOXJ1 protein expression in cells transfected with PBAE-formulated *FOXJ1* mRNA, whereas naked *FOXJ1* mRNA and PBAE-formulated GFP mRNA did not generate detectable FOXJ1 signal (Fig. 2A; Fig. S3). Across the doses tested, 100 ng of *FOXJ1* mRNA resulted in the highest proportion of FOXJ1-positive cells (50.9±19.2%) at 12 h post-transfection, compared with 14.9±4.9% and 3.6±1.3% for 50 and 200 ng, respectively (****P<0.0001; Fig. 2C,D). Naked *FOXJ1* mRNA produced no detectable FOXJ1-positive cells at any time point, consistent with inefficient uptake in the absence of PBAE formulation (Fig. 2A,D). The percentage of FOXJ1-positive cells reduced to 5.4±4.7% at 48 h post-transfection with 100 ng, due to the transient activity of mRNA (Fig. 2D). Across the tested dose range, no significant increase in cytotoxicity was observed relative to non-transfected controls (Fig. S4).

Following confirmation of successful *FOXJ1* mRNA transfection and translation, which was optimal at the 100 ng dose in this study, we then examined the early transcriptional response to transient *FOXJ1* expression in submerged primary HBECs, a condition that does not itself support mucociliary differentiation but can give an indication of functional *FOXJ1* activity. The expression of selected *FOXJ1* downstream genes involved in ciliogenesis was measured by qPCR at 24 h post-transfection (Fig. 2E). *FOXJ1* mRNA transfection significantly induced transient expression of *DNALI1* (*P=0.03) and *RSPH9* (***P=0.0004), while the other genes examined, including *CETN2*, *DNAH11*, *RFX3*, *RFX2*, *TUBA1A*, *DYNC2H1*, and *IFT172*, showed modest changes that did not reach statistical significance (Fig. 2E).

Control transfections using naked *FOXJ1* mRNA, PBAE-formulated GFP mRNA, or PBAE alone did not induce these transcripts (Fig. S5). This confirmed that the observed transcriptional changes were mediated by *FOXJ1* expression rather than the transfection process or the PBAE vector itself. Together, these data show that PBAE-mediated *FOXJ1* mRNA transfection of submerged primary HBECs produces transient *FOXJ1* expression and induction of *FOXJ1*-associated ciliogenesis transcripts.

### PBAE-mediated *FOXJ1* mRNA transfection induces early activation of ciliogenesis-related genes while preserving epithelial morphology and junctional gene expression at ALI

**Fig. 3.**
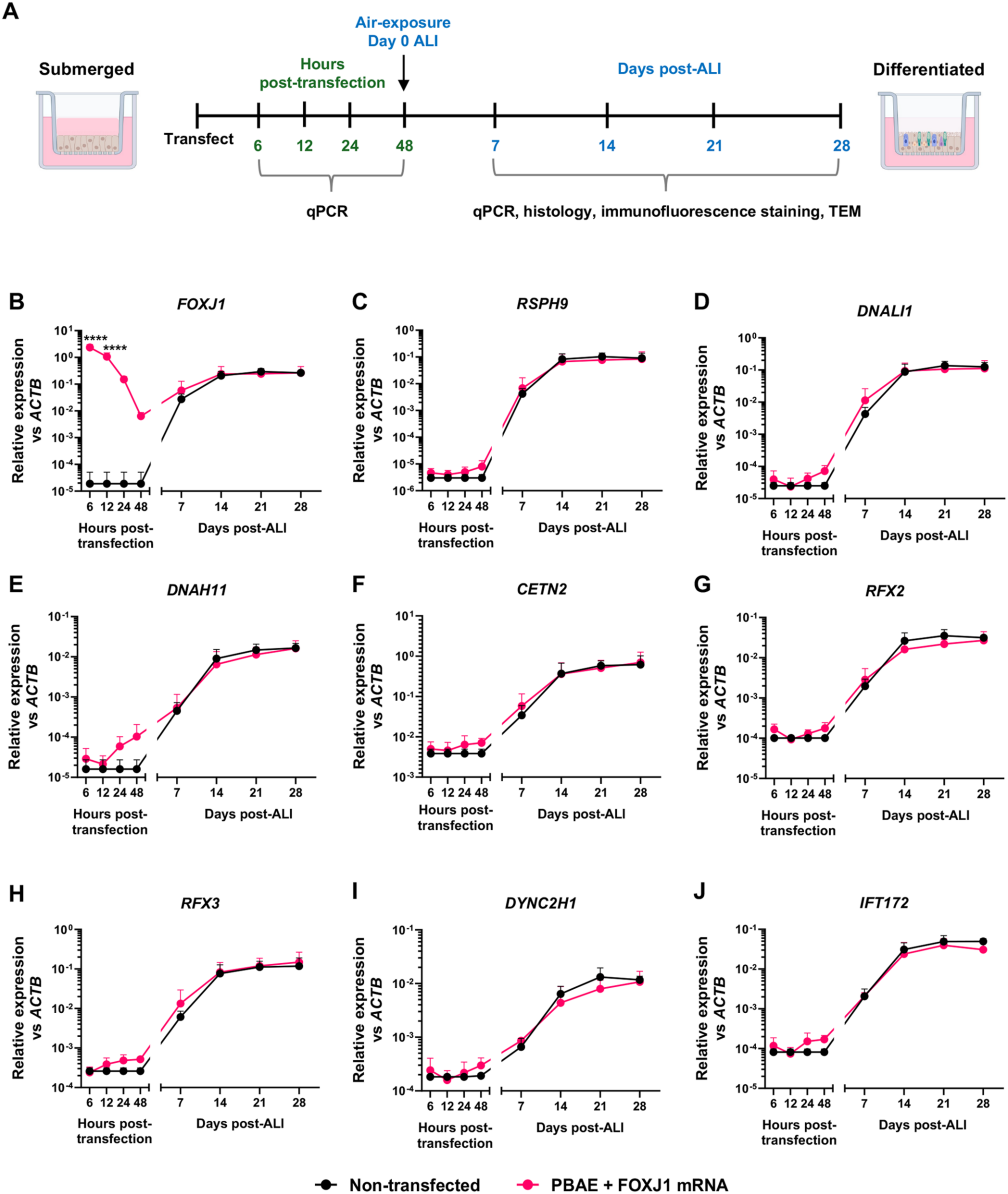
**PBAE-mediated *FOXJ1* mRNA transfection prior to ALI does not impair ciliogenesis-associated transcriptional programme of primary HBECs cultured at ALI.** (A) Experimental design for *FOXJ1* mRNA transfection. Primary HBECs were transfected with 100 ng *FOXJ1* mRNA 24 h after seeding onto Transwells, complexed with PBAE. Cells were harvested at 6, 12, 24, and 48 h post-transfection for qPCR analysis of *FOXJ1* downstream gene expression. Once *FOXJ1*-mRNA-transfected and non-transfected control cells formed a confluent monolayer, they were air-exposed and maintained at ALI for 28 days. Cells were harvested at days 7, 14, 21, and 28 post-ALI for qPCR, histological analysis, immunofluorescence staining, and TEM. (B-J) Expression of *FOXJ1* and its downstream ciliogenesis-associated genes at 6, 12, 24, and 48 h following *FOXJ1* mRNA transfection and at days 7, 14, 21, and 28 post-ALI. Data are presented as log2 fold change relative to non-transfected controls (*N*=3 donors, *n*=3 technical replicates, two-way ANOVA followed by Dunnett's multiple comparisons test; B, ****P<0.0001; C-J, non-significant *P*>0.9999).

The ALI culture system is a well-established *in vitro* model that closely recapitulates the *in vivo* airway environment by exposing the apical surface of cells to air while maintaining the basal surface in liquid (Fulcher et al., 2005; Pezzulo et al., 2011; Leung et al., 2020). Under these conditions, primary HBECs undergo mucociliary differentiation, acquiring key structural and functional characteristics of the native airway epithelium, including motile cilia formation, mucus-secreting cells, and tight junctions. To determine whether PBAE-mediated *FOXJ1* mRNA transfection before airlift altered subsequent differentiation at the ALI, primary HBECs were transfected with PBAE-formulated *FOXJ1* mRNA 24 h after seeding onto Transwell inserts (Fig. 3A). The apical medium was removed 48 h after transfection to establish ALI conditions (day 0 ALI), and *FOXJ1*-mRNA-transfected and non-transfected control HBECs were maintained for 28 days. qPCR confirmed efficient transfection prior to airlift, with *FOXJ1* mRNA being detectable at 6, 12, 24 and 48 h post-transfection compared to non-transfected controls (****P<0.0001) (Fig. 3B). In contrast, endogenous *FOXJ1* expression increased after airlift, and levels were similar in transfected and non-transfected cultures over the 28 days, consistent with ALI conditions primarily driving differentiation (Fig. 3B).

To assess whether transient *FOXJ1* expression prior to airlift was accompanied by changes in ciliogenesis-associated transcripts, the expression of a panel of *FOXJ1*-regulated genes was quantified across the ALI differentiation time course. During the 6- to 48-h submerged post-transfection period, several ciliary genes, including *RSPH9*, *DNALI*, *DNAH11*, *CETN2*, *RFX2*, *RFX3*, *DYNC2H1*, and *IFT172*, increased in expression, compared to the non-transfected control. However, these levels remained non-significant and lower than the transcriptional changes driven by ALI culture (Figs 3C-J). Although *DNALI1* and *RSPH9* were significantly upregulated at 24 h post-transfection in the 96-well plate format shown in Fig. 2E, these changes did not reach statistical significance in the independent Transwell-based experiments shown in Fig. 3. This likely reflects differences in culture format, including cell density, epithelial organisation, media volume and transfection efficiency, and therefore fold changes should be interpreted within each experimental set-up.

**Fig. 4.**
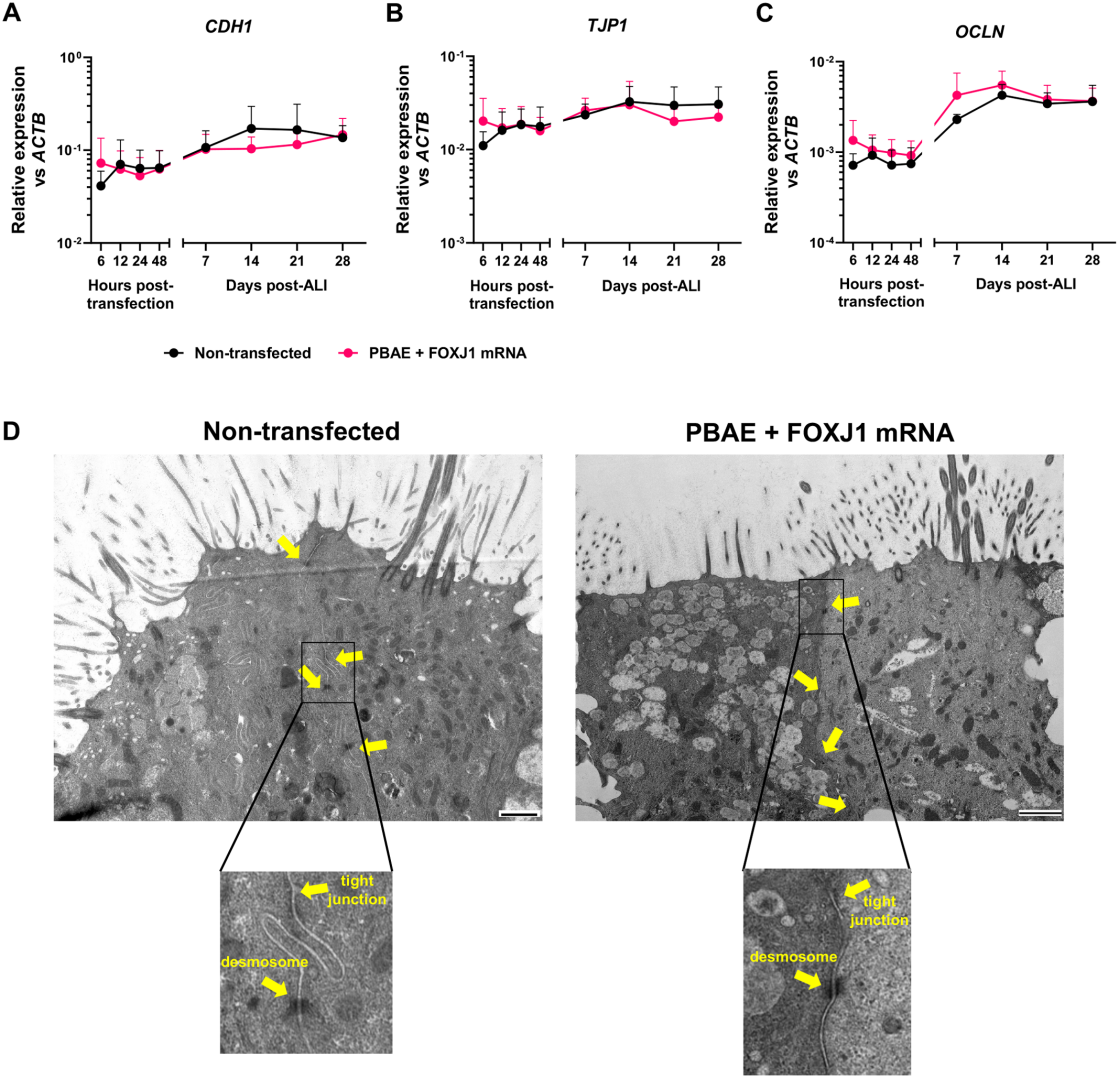
**PBAE-mediated *FOXJ1* mRNA transfection prior to ALI does not compromise epithelial morphology and expression of adhesion-related genes in primary HBECs cultured at ALI.** Primary HBECs were transfected with *FOXJ1* mRNA complexed with PBAE prior to air exposure. Non-transfected control cells and *FOXJ1*-mRNA-transfected cells were harvested at 6, 12, 24, and 48 h post-transfection to assess expression of epithelial integrity genes using qPCR. Once a confluent monolayer was established, cells were air-exposed and maintained at ALI for 28 days. Cells were further harvested at days 7, 14, 21, and 28 post-ALI for qPCR analysis of epithelial integrity markers. (A-C) Relative expression of *CDH1*, *TJP1*, and *OCLN* normalised to the housekeeping gene *ACTB* (mean±s.d.) (*N*=3 donors, *n*=3 technical replicates, two-way ANOVA followed by Dunnett's multiple comparisons test, non-significant *P*>0.9999). (D) Representative TEM images showing junctional complexes (yellow arrows) in non-transfected and *FOXJ1*-mRNA-transfected HBECs after 21 days of ALI culture. Scale bars: 6 µm.

We then asked whether PBAE-mediated *FOXJ1* mRNA transfection affected epithelial-barrier-associated markers. The expression of the adherens junction marker *CDH1* and the tight junction markers *TJP1* and *OCLN* was comparable between *FOXJ1*-mRNA-transfected and non-transfected cultures across multiple time points, including at 6, 12, 24, and 48 h post-transfection during submerged conditions, as well as days 7, 14, 21, and 28 post-ALI (Fig. 4A-C). Transmission electron microscopy (TEM) further demonstrated the presence of morphologically intact tight junctions and desmosomes in both groups (Fig. 4D). Together, these findings indicate that PBAE-mediated *FOXJ1* mRNA transfection of primary HBECs before airlift does not disrupt epithelial junctional gene expression or ultrastructural barrier features during ALI differentiation.

### Primary HBECs retain their capacity to differentiate at the ALI following PBAE-mediated *FOXJ1* mRNA transfection

**Fig. 5.**
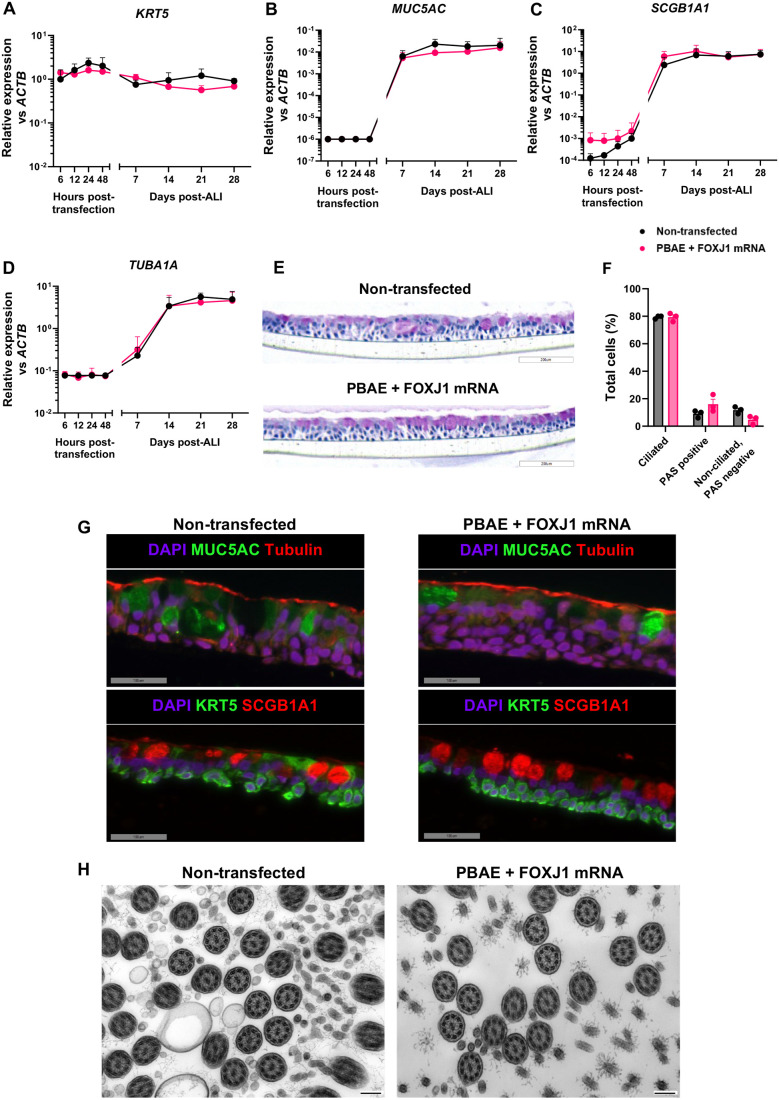
**Primary HBECs retain their capacity to differentiate at ALI following PBAE-mediated *FOXJ1* mRNA transfection.** Primary human HBECs were transfected with 100 ng of *FOXJ1* mRNA complexed with PBAE before air exposure. Cells were harvested at 6, 12, 24, and 48 h post-transfection for qPCR analysis of differentiation-related gene expression during submerged conditions. Once a confluent monolayer was established, cells were air-exposed and maintained at ALI for 28 days. Additional samples were harvested at days 7, 14, 21, and 28 post-ALI for qPCR analysis, histology, immunofluorescence and TEM characterisation. (A-D) Expression of *KRT5*, *MUC5AC*, *SCGB1A1* and *TUBA1A* normalised to the housekeeping gene *ACTB* (mean±s.d.) (*N*=3 donors, three technical replicates, two-way ANOVA followed by Dunnett's multiple comparisons test, non-significant *P*>0.9999). (E) Representative images of PAS staining of sections from non-transfected and *FOXJ1*-mRNA-transfected HBECs at 28 post-ALI. Scale bars: 200 μm. (F) Quantification of ciliated cells, PAS-positive goblet cells and non-ciliated PAS-negative cells in PAS-stained sections at day 28 post-ALI cultures, expressed as the percentage of total cells (mean±s.d.) (*N*=3 donors, with three technical replicates each, two-way ANOVA followed by Dunnett's multiple comparisons test, non-significant *P*>0.9999). (G) Representative immunofluorescence images for goblet cells (MUC5AC), cilia (acetylated alpha-tubulin), basal cells (KRT5), and club cells (SCGB1A1). Scale bars: 100 μm. (H) TEM assessment of cilia ultrastructure in non-transfected and *FOXJ1*-mRNA-transfected HBEC ALI cultures. Representative micrographs show transverse sections of cilia with the ‘9+2’ axoneme arrangement in day 28 post-ALI cultures. Scale bars: 200 nm.

To assess whether *FOXJ1* mRNA transfection affects the capacity of primary HBECs to undergo mucociliary differentiation during ALI culture, we measured the expression of epithelial-cell-type markers over time up to 28 days. Markers representing distinct epithelial populations were assessed, including *KRT5* for basal cells, *TUBA1A* for cilia, *MUC5AC* for goblet cells, and *SCGB1A1* for club cells. With the exception of *KRT5*, all differentiation-associated markers increased over the course of ALI culture, peaking between days 7 and 21 post-ALI before plateauing (Fig. 5A-D). *SCGB1A1* had a slight increase at early time points pre-airlift in *FOXJ1*-mRNA-transfected HBECs compared to controls; however, this was insignificant and converged to levels seen in control cultures post-airlift (Fig. 5C). There was no difference in temporal expression of *KRT5*, *MUC5AC* and *TUBA1A* between groups during differentiation (Fig. 5A,B,D).

Histological assessment at day 28 post-ALI showed that both groups formed a comparable pseudostratified mucociliary epithelium (Fig. 5E). Quantification of cell populations showed a similar overall composition between groups, with ciliated cells comprising 79.3% of the total population in both conditions (Fig. 5F). The proportions of periodic acid-Schiff (PAS)-positive cells (9% in non-transfected HBECs versus 16% in *FOXJ1*-mRNA-transfected HBECs) and PAS-negative non-ciliated cells (11.7% in non-transfected HBECs versus 4.7% in *FOXJ1*-mRNA-transfected HBECs) were also comparable between groups (*P*>0.99) (Fig. 5F). End-point immunofluorescence analysis at day 28 post-ALI confirmed the presence of MUC5AC-positive goblet cells, acetylated alpha-tubulin-positive cilia, KRT5-positive basal cells, and SCGB1A-positive club cells in both groups (Fig. 5G).

To determine whether cilia ultrastructure was preserved, TEM was performed on day 28 post-ALI cultures. Both *FOXJ1*-mRNA-transfected and non-transfected HBECs displayed well-organised cilia with the expected 9+2 axonemal arrangement, intact microtubules, radial spokes, and dynein arms (Fig. 5H). Overall, these findings demonstrate that PBAE-mediated *FOXJ1* mRNA transfection before ALI establishment does not alter the time course of differentiation, final epithelial cell phenotype, or ciliary ultrastructure.

## DISCUSSION

This study examined PBAE-mediated delivery of IVT-mRNA to primary basal HBECs and the impact of this intervention on subsequent differentiation under ALI conditions. Using GFP reporter mRNA, we first established PBAE-mediated transfection in primary basal HBECs and demonstrated that at day 28 of ALI, epithelial differentiation and barrier integrity were maintained. We then used *FOXJ1* IVT-mRNA as a biologically relevant transcription factor cargo to assess in more detail the impact of transfection and whether the transient expression of mRNA could activate expected downstream transcriptional targets without disrupting epithelial differentiation. To the best of our knowledge, this approach has not previously been demonstrated in primary HBECs. Our data demonstrated that PBAE-mediated transfection enabled efficient delivery and translation of exogenous *FOXJ1* mRNA, resulting in transient upregulation of ciliogenesis-associated genes without compromising their capacity for mucociliary differentiation. ALI-driven epithelial differentiation, lineage composition, and ciliary ultrastructure were preserved in *FOXJ1*-mRNA-transfected cells, an important consideration when applying novel gene modulation strategies to progenitor cell populations.

The ALI culture system provides a physiologically relevant platform to explore novel mRNA-based interventions, as it enables basal HBECs to differentiate into a pseudostratified mucociliary epithelium. Previous studies have used ALI cultures to screen nanoparticle formulations (Jiang et al., 2024) and evaluate candidate therapeutic targets relevant to disorders such as PCD (Hennig et al., 2025). However, these approaches have largely focused on delivery to established ALI cultures. This is the first study to detail the impact of a model mRNA-encoded transcription factor on primary basal HBEC differentiation. The custom-designed *FOXJ1* IVT-mRNA used here incorporated key modifications, including a Cap 1 structure, polyadenylated tail, and pseudouridine nucleotides, which are commonly used in clinically approved mRNA medicines to enhance translational efficiency and reduce innate immune activation (Gallie, 1991; Karikó et al., 2005, 2008; Nicholson and Pasquinelli, 2019). This was combined with a polymeric nanoparticle platform previously shown to enable nebulised mRNA delivery in preclinical mouse models (Patel et al., 2019; Blanchard et al., 2021; Vanover et al., 2022; Jiang et al., 2024).

A transient increase in the proportion of FOXJ1-positive cells was observed following mRNA transfection, peaking at 12 h and declining by 48 h, consistent with the expected kinetics of IVT-mRNA expression and previous studies using mRNA-encoded transcription factors (Ma et al., 2020; Qabrati et al., 2023). Although delivery of a higher mRNA dose by PBAE was associated with increased *FOXJ1* mRNA abundance compared to naked mRNA, this did not translate into a proportional increase in the proportion of FOXJ1-positive cells. This indicates that although mRNA uptake was dose responsive, the percentage of cells transfected was not. The lack of an increase in the number of cells expressing FOXJ1 protein at the higher dose may be due to limitations in methodology, such as the absence of protein-level measurements, or to challenges in endosomal trafficking, intracellular degradation or translational capacity, although these mechanisms were not directly tested here. These results emphasise the importance of optimising mRNA dose and formulation based on protein-level readouts and cell viability, rather than mRNA abundance alone.

Given the complexity of the *FOXJ1*-regulated ciliogenic programme, gene expression analysis focused on representative markers from three key functional categories: (1) cilia-related transcription factors (*RFX3*, *RFX2*); (2) structural and motility-associated genes (*CETN2*, *DNAH11*, *DNALI1*, *RSPH9*, *TUBA1A*); and (3) intraflagellar transport components (*DYNC2H1*, *IFT172*). *FOXJ1* mRNA transfection led to transient upregulation of several ciliogenesis-related genes under submerged conditions, suggesting that translated FOXJ1 protein was active and capable of engaging part of the expected transcriptional programme. These findings extend previous work by Didon et al. (2013), who demonstrated that FOXJ1 overexpression in basal cells by DNA-based approaches can induce similar cilia-associated genes, including *CETN2*, *DNAH11*, dynein axonemal intermediate chain 1 (*DNAI1*), *DNALI1*, EF-hand domain C-terminal containing 1 (*EFHC1*), sperm-associated antigen 6 (*SPAG6*), Tektin 1 (*TEKT1*), *TEKT2*, and *TUBA1A2*. While *FOXJ1* mRNA transiently activated ciliogenesis-associated transcripts before airlift, these levels were relatively low compared to levels induced by airlift differentiation. This was not surprising, as a single transient *FOXJ1* mRNA transfection is not sufficient to drive differentiation as previously reported. Additional differentiation cues, prolonged *FOXJ1* expression, and/or combinatorial approaches with targeted pathway modulation may be required to produce stronger or more durable effects (Gerovac et al., 2014; Dreyer et al., 2026).

Maintaining epithelial barrier integrity and appropriate lineage composition is essential for mucociliary function. Histological, transcriptional, and ultrastructural assessments did not indicate disruption of epithelial differentiation following *FOXJ1* mRNA transfection. Transfected cultures formed a pseudostratified epithelium containing ciliated and secretory cells comparable to controls, and junctional marker expression (*TJP1*, *OCLN*, *CDH1*) was maintained across timepoints. TEM further supported preserved junctional architecture and showed no obvious abnormalities in ciliary ultrastructure, with the typical 9+2 axonemal organisation observed in both groups.

Despite these encouraging findings, certain limitations should be acknowledged. Donor numbers were limited, underscoring the need for larger cohorts to evaluate inter-donor variability in transfection efficiency and downstream transcriptional responses. In addition, the transient expression window following a single IVT-mRNA dose likely constrained the magnitude and duration of the observed transcriptional effects. The impact of repeated dosing on differentiation should be further investigated (Qabrati et al., 2023). Although ultrastructural data supported preserved cilia architecture, functional readouts such as ciliary beat frequency and mucociliary transport measurements would be valuable to determine whether transient FOXJ1 expression alters ciliary function.

In conclusion, PBAE-mediated delivery of *FOXJ1* IVT-mRNA achieved efficient, transient FOXJ1 expression in primary HBECs and induced an early induction of ciliogenesis-related transcripts, demonstrating that non-viral mRNA delivery of a transcription factor of interest can activate downstream gene programmes in this physiologically relevant human bronchial epithelial model. Importantly, transient transfection did not impede epithelial integrity, differentiation, or ciliary ultrastructure during ALI culture. Collectively, our findings support the use of the primary human ALI as a platform for evaluating transient mRNA-based gene modulation in airway epithelial cells.

## MATERIALS AND METHODS

### Primary HBEC culture

Primary HBECs from healthy donors were obtained from Lonza (CC-2540S, Basel, Switzerland) or Epithelix (EP51AB, Geneva, Switzerland) and cultured in airway epithelial growth medium (Promocell) supplemented as directed by the supplier. Cultures were maintained at 37°C in a 5% CO₂ incubator. Cryopreserved HBECs were thawed and seeded into T75 collagen-coated flasks (PureCol^®^ bovine type I collagen solution, 30 µg/ml, Advanced BioMatrix). The medium was replaced 24 h after seeding and subsequently every 48-72 h. Cells were passaged at 70-80% confluence by washing with PBS, detaching with TrypLE™ Express Enzyme (Gibco), centrifuging, and resuspending in fresh medium. Cell viability and counts were determined using Trypan Blue (Gibco).

For ALI cultures, HBECs were seeded at 30,000 cells per collagen-coated Transwell insert (Greiner Bio-One) in a 1:1 mixture of airway epithelial growth medium (Promocell) and DMEM with GlutaMAX™ (Gibco), supplemented as directed by the supplier, excluding triiodo-L-thyronine. The following supplements were also added: HEPES (25 mM) (Gibco) and BSA (1.5 µg/ml) (Sigma-Aldrich). Upon reaching confluence, the apical medium was removed to expose the cells to air, while a PneumaCult™ ALI medium (STEMCELL) supplemented with heparin (0.0004%) (STEMCELL) and hydrocortisone (0.48 µg/ml) (STEMCELL) was maintained in the basal compartment and refreshed every 2-3 days. Differentiation was confirmed by observing cilia beating and mucus production using light microscopy (Stölting et al., 2022).

### PBAE synthesis and characterisation

PBAE was synthesised as previously described (Patel et al., 2019). Briefly, a branched DD90-118 (PBAE) polymer was synthesised by reacting bisphenol glycerolate (diacrylate DD) with 4-(2-aminoethyl) morpholine (backbone amine 90) and *N*-methyl 1,3-diaminopropane (branching amine) (Sigma-Aldrich) at a 1:0.5:0.2 molar ratio in anhydrous dimethylformamide for 48 h. After cooling to 30°C, end-cap amine (1,5-diamino-2-methylpentane) (118) (Sigma-Aldrich) was added at a molar equivalent to the excess acrylate and stirred for an additional 24 h at 30°C. The polymer was purified in cold diethyl ether and dried under vacuum for 48 h at room temperature and stored at −80°C.

### mRNA transfection of primary HBECs

Twenty-four hours before transfection, cells were seeded in 96-well plates coated with PureCol^®^ bovine type I collagen solution (30 µg/ml), Transwell inserts, or eight-well chamber slides using the airway epithelial growth medium described above. Seeding densities were 12,000 cells/well (100 µl) for 96-well plates, 30,000 cells/insert (200 µl) for Transwell inserts, and 50,000 cells/well (200 µl) for eight-well chamber slides. Fresh medium was added before transfection. Cy-tagged GFP mRNA (7701-100; Cap 1, polyadenylated, 5moU) was purchased from Trilink, USA). *FOXJ1* mRNA (Ref Seq: NM_001454.4, Gene ID: 2302) (Cap 1, polyadenylated, 5moU) was purchased from OZ Biosciences, France. PBAE stock solution (50 mg/ml in DMSO) was diluted in 100 mM sodium acetate buffer (pH 5.2, NaOAc) (Sigma-Aldrich) to allow equal-volume complexation of PBAE:mRNA at a mass ratio of 50:1 and diluted in PBS to achieve a final NaOAc concentration of 12.5 mM. Complexes were mixed by pipetting and incubated at room temperature for 10 min. For naked mRNA transfections, mRNA was diluted in sterile nuclease-free water. Vector-only controls were prepared similarly using nuclease-free water instead of mRNA. For 96-well plates or eight-well chamber slides, a total volume of 15 or 30 µl containing the desired mRNA dose was added per well, respectively, and the medium was changed after 4 h. Cells were incubated at 37°C with 5% CO_2_ until further assays.

### Characterisation of PBAE-mRNA complexes

Particle size and zeta potential of PBAE complexed with mRNA were determined by dynamic light scattering (DLS) using a Zetasizer NanoZSP (Malvern Instruments). Particles were diluted 1:10 in deionised water, with 100 µl analysed in sizing cuvettes (VWR) or 600 µl in zeta cuvettes (Malvern).

To visually assess mRNA entrapment, PBAE-mRNA particles were loaded onto an Egel™ Ex 2% Agarose Gel (Invitrogen) with the Riboruler™ High RNA ladder (Thermo Fisher Scientific) as a reference. Electrophoresis was conducted for 15 min using the E-Gel™ Power Snap System (Invitrogen), followed by imaging with the iBright 1500 system (Invitrogen). For quantification of mRNA encapsulation efficiency, the Quant-iT™ RiboGreen™ RNA Kit (Invitrogen) was used with slight modifications to the manufacturer's protocol. A Tris-EDTA (TE) working solution was prepared by 20-fold dilution of the concentrated buffer with nuclease-free water. The Quant-iT™ RiboGreen™ RNA reagent was diluted 200-fold with the TE working solution. A standard curve was generated using a non-encapsulated mRNA solution at 2 µg/ml in TE. Particles, prepared as previously described to a final mRNA concentration of 2 µg/ml, were diluted in TE to 500 µl, mixed 1:1 with the Quant-iT™ RiboGreen™ RNA reagent working solution, and centrifuged at 8000***g*** for 5 min. One hundred microlitres of the supernatant was transferred to a black-bottom 96-well plate, and fluorescence was measured using a SpectraMax® i3 plate reader (Molecular Devices) at 480 nm excitation and 520 nm emission. The maximum fluorescence from free mRNA represented 0% encapsulation efficiency. Encapsulation efficiency was calculated using the following formula:

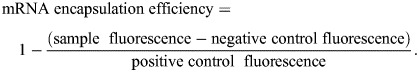


### LDH cytotoxicity assay

Cellular cytotoxicity was evaluated using the LDH assay (Roche) according to the manufacturer's protocol. Culture supernatants were collected, combined with a reaction mixture, and incubated for 10 min at 37°C. Absorbance was measured at 490 nm using a SpectraMax^®^ i3 plate reader (Molecular Devices). Controls included a positive control (cells treated with 1% Triton X-100 for maximum LDH release), a negative control (medium alone), and a spontaneous-release control (untreated cells). Cytotoxicity, expressed as the percentage of LDH release relative to the positive control, was calculated using the following formula:

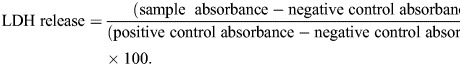


### Gene expression analysis

RNA was isolated from cells using the RNeasy Micro Plus kit (Qiagen), and its concentration and purity were assessed using a NanoDrop™ 1000 spectrophotometer (Thermo Fisher Scientific). For cDNA synthesis, 10 µl of RNA per reaction was reverse-transcribed using the High-Capacity cDNA Reverse Transcription Kit (Thermo Fisher Scientific) in a 20 µl reaction, following the manufacturer's protocol. mRNA expression was quantified by real-time qPCR (RT-qPCR) using TaqMan™ FAM assays (Thermo Fisher Scientific) and Fast Advanced Master Mix (Thermo Fisher Scientific) on a ViiA™ 7 Real-Time PCR System (Thermo Fisher Scientific) in duplicate in 384-well plates. The following TaqMan™ gene expression assays were used: *ACTB* (Hs01060665_g1), *SCGB1A1* (Hs00171092_m1), *KRT5* (Hs00361185_m1), *FOXJ1* (Hs00230964_m1), *MUC5AC* (Hs01365616_m1), *TJP1* (Hs01551861_m1), *CDH1* (Hs01023895_m1), *OCLN* (Hs05465837_g1), *CETN2* (Hs00942570_g1), *DNAH11* (Hs00361951_m1), *DNALI1* (Hs00185750_m1), *DYNC2H1* (Hs00300261_m1), *RFX2* (Hs00172177_m1), *RFX3* (Hs00231292_m1), *RSPH9* (Hs01040988_m1), and *TUBA1A* (Hs03045184_g1). Relative expression was calculated using the 2^−ΔCT^ method (where ΔCT=CT gene of interest−CT housekeeping gene), with *ACTB* as the housekeeping gene.

### Histology processing of HBEC ALI cultures

Primary HBECs cultured on Transwell inserts were fixed in 4% paraformaldehyde (PFA; Thermo Fisher Scientific) in PBS for 20 min at room temperature and then washed three times with PBS. A 4% low-melting-point agarose solution (Thermo Fisher Scientific) was prepared by dissolving agarose in PBS, heating, and then cooling to 37°C. Membranes were excised from Transwell inserts using a scalpel and individually embedded in the agarose solution placed in a 24-well plate, ensuring they remained flat and fully submerged. The agarose was solidified on ice before the embedded membranes were transferred to histology cassettes. Samples were dehydrated through a graded ethanol series (30%, 50%, and 70% for 15 min each) and then submitted to the Histology Core Facility for paraffin embedding, 4 µm sectioning, and PAS staining. Stained cross sections were imaged using an Aperio VERSA 8 slide scanner microscope (Leica). The numbers of ciliated cells and PAS-positive goblet cells in PAS-stained sections were quantified using the Aperio ImageScope Software (Leica, version 12.4.6.5003).

### TEM

Primary HBECs cultured at ALI were fixed in 2.5% glutaraldehyde (Merck) in PBS for 1 h at room temperature and then submitted to the Electron Microscopy Unit at Royal Brompton Hospital. Samples were immersed in 0.05 M sodium cacodylate buffer (Agar Scientific) and processed within 2 weeks. They underwent a 1-h post-fixation with 1% osmium tetroxide (TAAB) in cacodylate buffer at room temperature, followed by rinsing with purified water and dehydration through an ascending ethanol series (70%, 90%, and 100%). Samples were transitioned to 100% Araldite CY212 (Agar Scientific) using an ethanol:Araldite gradient (50:50, 25:75 mixtures) before overnight immersion in 100% Araldite. They were then embedded in moulds with fresh Araldite and polymerised at 60°C for 3 days. Araldite-embedded blocks were sectioned at 1 µm thickness, stained with Toluidine Blue (Agar Scientific), and inspected by light microscopy to select regions of interest (ROIs). Blocks were trimmed to the ROIs, ultrathin sectioned at 80 nm, and mounted on 200-mesh copper grids for TEM. Ultrathin sections were contrast-stained with 1.5% uranyl acetate (Agar Scientific) in methanol, followed by Reynolds lead citrate solution (Agar Scientific). Sections were examined with a TEM at 120 KV (JEOL 1400+), and micrographs were captured at magnifications from ×10,000 to ×1,000,000 for morphological assessments (using an AMT XR16 camera).

### Immunofluorescence staining and imaging

Cells were fixed with 4% PFA in PBS for 20 min at room temperature, washed with PBS, and stored in PBS at 4°C until staining. Submerged cells on eight-well chamber slides were permeabilised and blocked with 10% species-appropriate serum (goat serum: Abcam, donkey serum: Abcam) and 0.1% Triton X-100 (Sigma-Aldrich) in PBS for 1 h at room temperature. Cells were incubated overnight at 4°C in 1% BSA with the following primary antibodies: acetylated alpha-tubulin (mouse, clone 6-11B-1; Abcam; 1:100), MUC5AC (rabbit polyclonal; Abcam; 1:100), KRT5 (rabbit, clone EP1601Y; Abcam; 1:100), ZO-1 (mouse, clone ZO1-1A12; Thermo Fisher Scientific; 1:100), FOXJ1 (goat polyclonal; R&D Systems; 1:100), and SCGB1A1 (rat, clone 394324; R&D Systems; 1:100). After washing, cells were incubated for 1 h at room temperature (protected from light) with fluorophore-conjugated secondary antibodies diluted in 1% BSA: goat anti-rabbit IgG (Alexa Fluor 488 or Alexa Fluor 555; Thermo Fisher Scientific; 1:500), goat anti-mouse IgG (Alexa Fluor 488 or Alexa Fluor 555; Thermo Fisher Scientific; 1:500), goat anti-rat IgG (Alexa Fluor 488 or Alexa Fluor 555; Thermo Fisher Scientific; 1:500), or donkey anti-goat IgG (NorthernLights™ 557; R&D Systems; 1:200). Cells were then washed, counterstained with DAPI (1:2000 in PBS) (Sigma-Aldrich) for 10 min in the dark and mounted with ProLong™ Diamond Antifade Mountant (Invitrogen).

For primary HBECs cultured at ALI (cross sections), paraffin-embedded sections were deparaffinised with D-limonene, rehydrated through an ethanol series, and subjected to heat-induced antigen retrieval in 90°C EDTA buffer (pH 8) for 30 min. Cross sections were then stained as described above and mounted with ProLong™ Diamond Antifade Mountant. Immunofluorescence images were acquired using a Leica SP5 inverted confocal microscope or an Aperio VERSA 8 slide scanner and processed with ImageJ. ImageJ's counting macro was used for nucleus quantification.

### TEER

TEER was measured in ALI cultures using Millicell^®^ ERS electrode probes (Millipore, MERSSTX01) connected to a Millicell^®^ ERS-2 voltohmmeter (Millipore, MERS00002), following the manufacturer's instructions. Prior to measurement, the electrodes were sterilised in 70% ethanol and rinsed with sterile PBS. For each Transwell insert, 100 µl of pre-warmed sterile 1× PBS was added to the apical chamber. To minimise variability due to temperature differences between the 37°C incubator and room temperature, cultures were allowed to equilibrate before measurements were taken. During measurement, the electrodes were positioned vertically in the apical and basal compartments of each Transwell. Blank inserts lacking cells were measured in parallel to determine background resistance. TEER value (Ω×cm^2^) was calculated using the following formula:

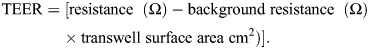


### Permeability assay

Permeability of ALI cultures was evaluated using a FITC-dextran permeability assay. Prior to the assay, the apical surface of each Transwell insert was washed with 200 µl of PBS. FITC-dextran (4 kDa; Sigma-Aldrich), prepared in sterile PBS at 1 mg/ml, was then added to the apical chamber (200 µl per insert), while the basal chamber was filled with 500 µl of PBS. The paracellular flux of FITC-dextran from the apical to the basal compartment was assessed after 30 min of incubation at 37°C in a humidified 5% CO_2_ incubator. Following incubation, 100 µl aliquots were collected from the basal chamber, transferred to a black-bottom 96-well plate, and fluorescence was measured using a SpectraMax^®^ i3 plate reader (Molecular Devices) at 490 nm excitation and 520 nm emission. FITC-dextran leakage from the apical to the basal chamber was calculated relative to an empty Transwell control using the following formula:

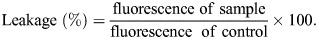


### Statistical analysis

Data analysis and graph generation were performed using GraphPad Prism (version 9.5.1). Results are presented as mean±standard deviation (s.d.). Detailed data representation, including the use of individual data points where applicable, is provided in the figure legends. The statistical test applied for each experiment is specified in the relevant figure legend. *P*-values<0.05 were considered statistically significant. The number of biological and technical replicates for each experiment is specified in the figure legends.

## Supplementary Material



10.1242/biolopen.062419_sup1 Supplementary information
