# Supplementary material for: Investigating the impact of poly(beta amino) ester-mediated FOXJ1 mRNA delivery on differentiation of primary human bronchial epithelial cells
Source: Biol Open. 2026 Jun 12;15(6):bio062419. doi: 10.1242/bio.062419 (PMC13312916; doi:10.1242/bio.062419)
Supplement: Supplementary information [file biolopen-15-062419-s1.pdf]

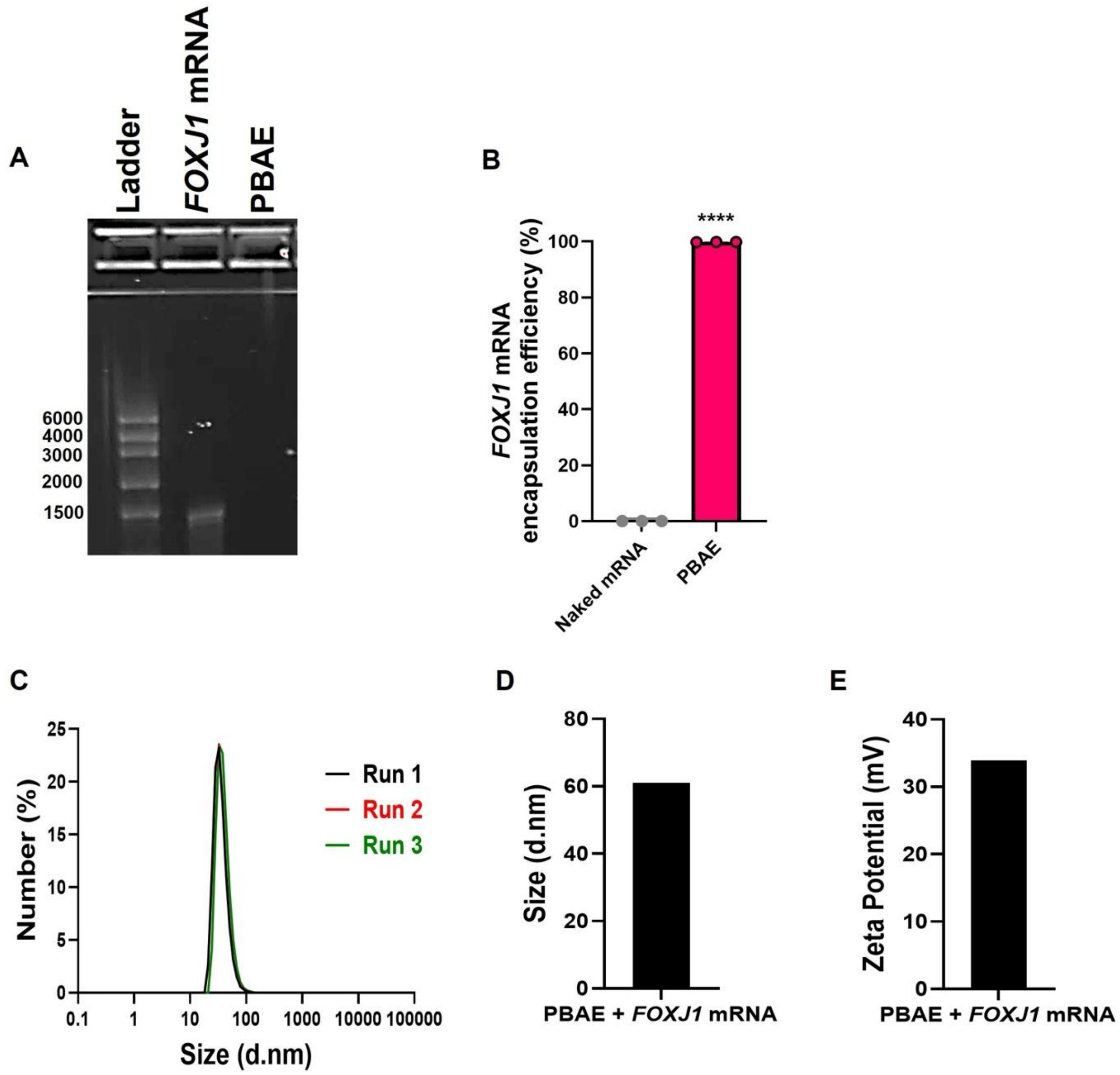

Fig. S1. Characterisation of PBAE/*FOXJ1* mRNA complex. (A) Gel retardation assay of PBAE/*FOXJ1* mRNA complex analysed via 2% agarose gel electrophoresis. (B) Encapsulation efficiency of *FOXJ1* mRNA complexed with PBAE, assessed using the RiboGreen assay. Measurements were performed in triplicate for each condition (mean ± SD). (Unpaired t-test; ****P<0.0001). (C) Size distribution of PBAE/*FOXJ1* mRNA complexes measured by DLS. (D) Particle size in nm for PBAE/*FOXJ1* mRNA complexes. (E) Zeta Potential measurement of PBAE/*FOXJ1* mRNA complexes.

Biology Open • Supplementary information

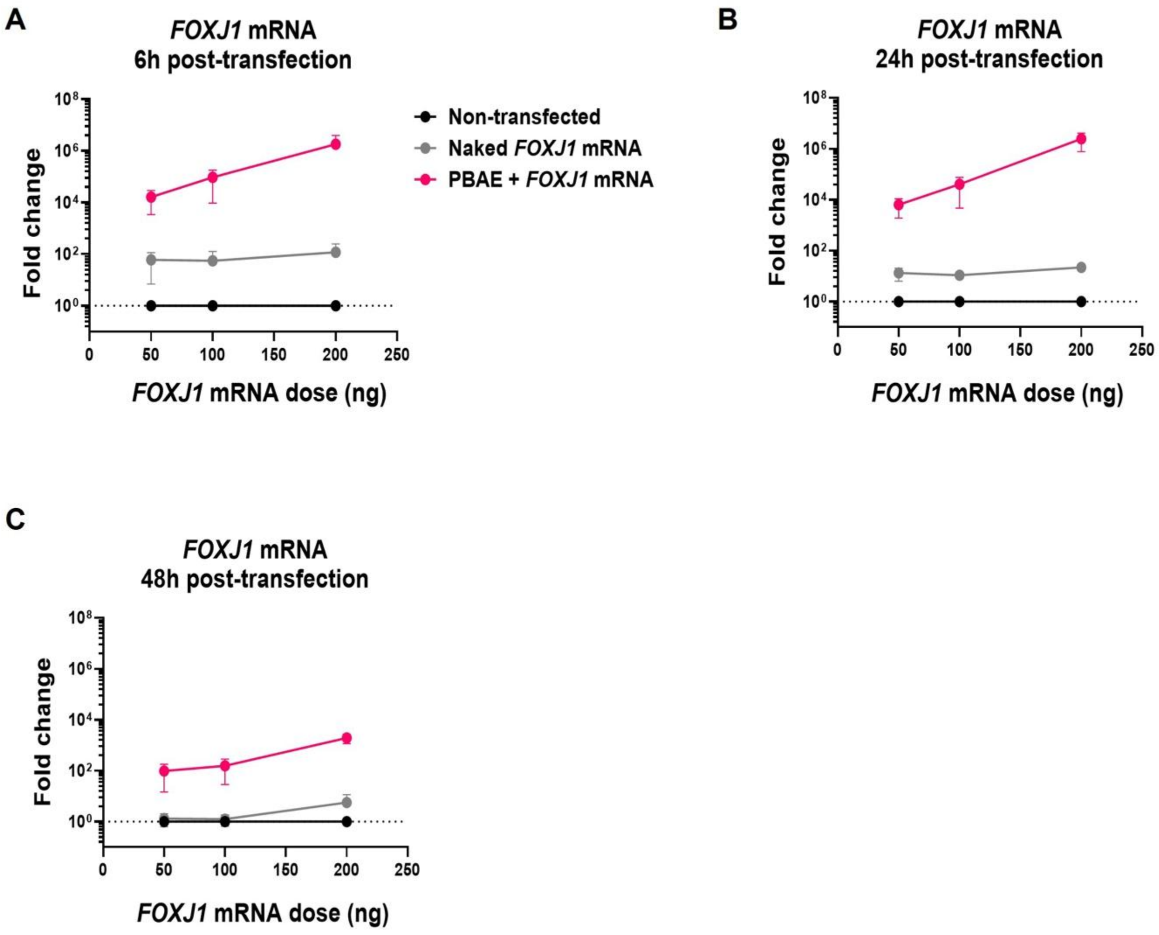

Fig. S2. Expression of *FOXJ1* following *FOXJ1* mRNA transfection in submerged primary HBECs. Submerged primary HBECs seeded on 96-well plates were transfected with increasing doses of *FOXJ1* mRNA (50/100/200 ng) delivered naked or complexed with PBAE. *FOXJ1* expression was quantified by qPCR at different time points post-transfection, expressed as fold change from non-transfected controls: (A) 6 hours, (B) 24 hours, (C) 48 hours (mean $\pm$ SD). N = 3 donors, n = 3 technical replicates (2way ANOVA followed by Dunnett's multiple comparisons test; non-significant P>0.9999).

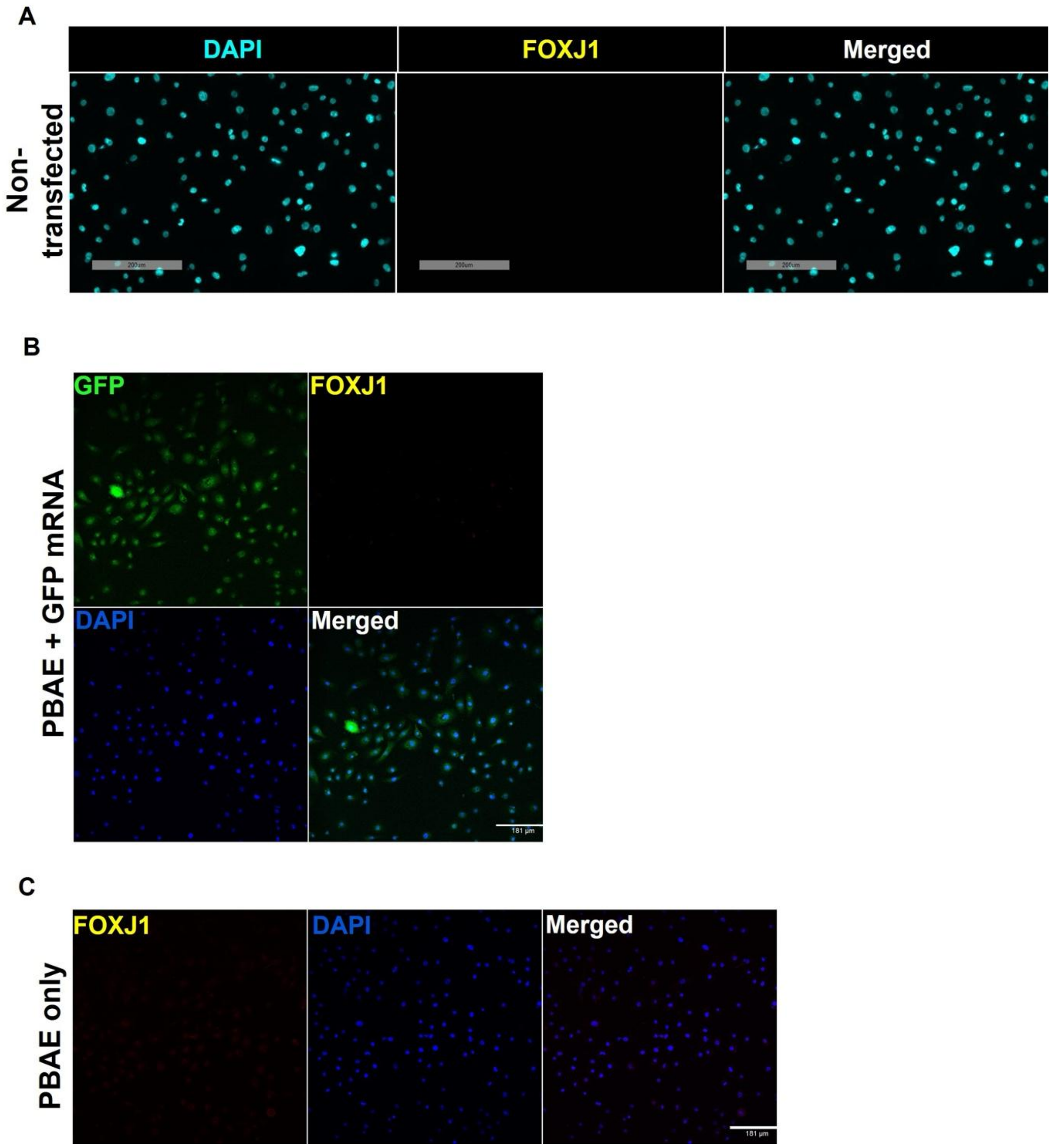

Fig. S3. Control transfections do not induce FOXJ1 protein expression in submerged primary HBECs. Submerged primary HBECs were seeded on 8-well chamber slides and imaged 12 hours post-transfection. Cells were stained with anti-FOXJ1 antibody and DAPI. (A) Non-transfected control HBECs. (B) HBECs transfected with GFP mRNA complexed with PBAE. (C) HBECs transfected with PBAE only control.

Biology Open • Supplementary information

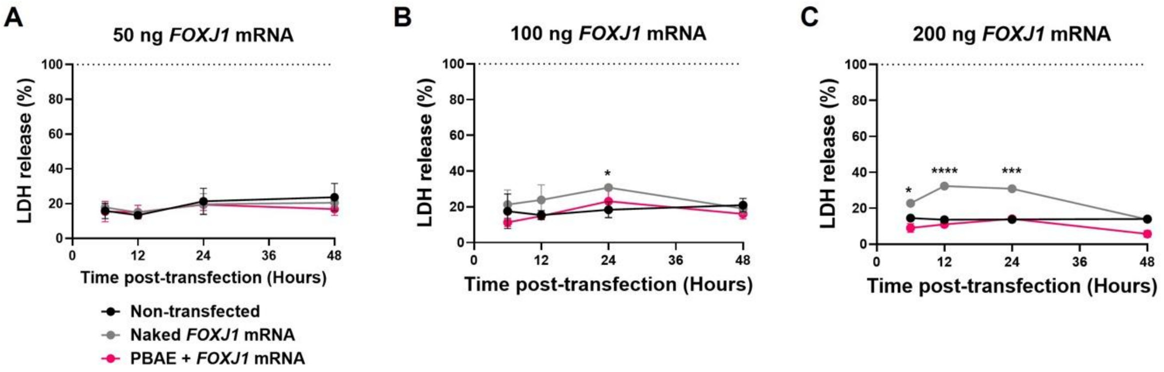

Fig. S4. Cytotoxicity following *FOXJ1* mRNA transfection of submerged primary HBECs. Submerged primary HBECs seeded on 8-well chamber slides were transfected with increasing doses of *FOXJ1* mRNA (50, 100, 200 ng) either delivered naked or complexed with PBAE. Cytotoxicity was assessed at 6, 12, 24, and 48 hours post-transfection by LDH release (%) relative to a 1% Triton-X-100 positive control. (A) 50 ng, (B) 100 ng, (C) 200 ng *FOXJ1* mRNA (mean ± SD). (N = 3 donors, n = 3 technical replicates, 50 ng FOXJ1 mRNA: 2way ANOVA followed by Dunnett's multiple comparisons test; non-significant P=0.75. 100 ng FOXJ1 mRNA: 2way ANOVA followed by Dunnett's multiple comparisons test; non-transfected vs Naked FOXJ1 mRNA at 24 hours post-transfection *P=0.0468, rest non-significant P=0.4399. 200 ng FOXJ1 mRNA: 2way ANOVA followed by Dunnett's multiple comparisons test; non-transfected vs naked FOXJ1 mRNA at 6 hours post-transfection *P=0.0115, non-transfected vs naked FOXJ1 mRNA at 12 hours post-transfection ****P<0.0001, non-transfected vs naked FOXJ1 mRNA at 24 hours post-transfection ***P=0.0001. Rest non-significant P=0.3).

Biology Open • Supplementary information

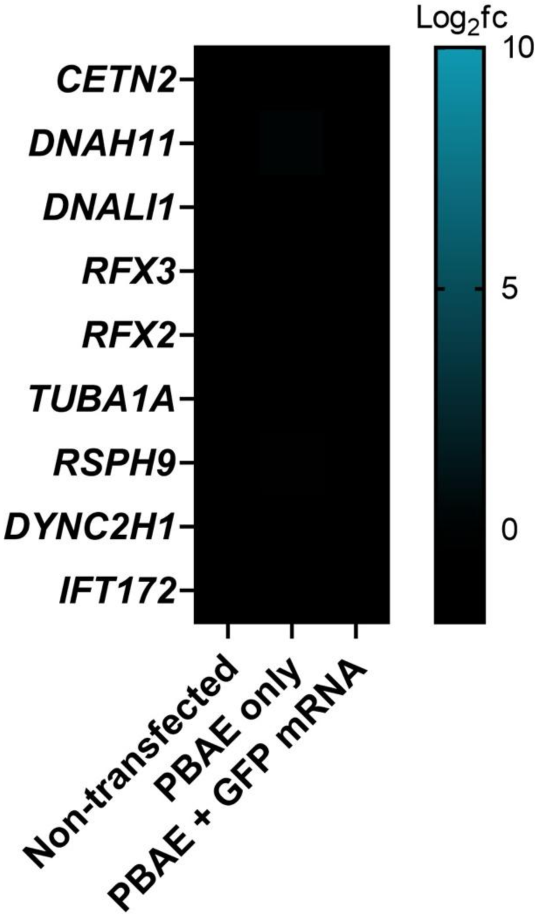

Fig. S5. Transfection with GFP mRNA/PBAE or PBAE alone does not induce ciliogenesis gene expression in primary HBECs. Submerged primary HBECs seeded on 96-well plates were transfected with 100 ng GFP mRNA complexed with PBAE or PBAE alone. qPCR analysis at 24 hours post-transfection showed no induction of ciliated cell-associated genes. Data are presented as log2 fold change relative to non-transfected controls. N = 3 donors, n = 3 technical replicates.

Biology Open • Supplementary information
