# Supplementary material for: Investigating the impact of poly(beta amino) ester-mediated FOXJ1 mRNA delivery on differentiation of primary human bronchial epithelial cells
Source: Biol Open. 2026 Jun 12;15(6):bio062419. doi: 10.1242/bio.062419 (PMC13312916; doi:10.1242/bio.062419)
Supplement: Supplementary file 1 [file biolopen-15-062419_review_history.pdf]

Investigating the impact of PBAE-mediated FOXJ1 mRNA delivery on differentiation of primary human bronchial epithelial cells

Rafaela Konstantinidi, Mira Subramanian, Laura Yates, Clare M. Lloyd, Sejal Saglani and Asha Kumari Patel
10.1242/bio.062419

Editor: Alissa Armstrong

Review timeline

Original submission:	12 December 2025
Editorial decision:	26 December 2025
First revision received:	20 April 2026
Editorial decision:	4 May 2026
Second revision received:	15 May 2026
Accepted:	17 May 2026

Original submission

First decision letter

MS ID#: bio.062419

MS Title: Investigating the impact of PBAE-mediated FOXJ1 mRNA delivery on differentiation of primary human bronchial epithelial cells

Authors: Rafaela Konstantinidi; Mira Subramanian; Laura Yates; Clare M Lloyd; Sejal Saglani; Asha Kumari Patel

I have now reached a decision on the above manuscript.

The reviewer reports are shown at the bottom of this email.

As you will see, the reviewers raised a number of substantial criticisms that prevent me from accepting the paper at this stage.

They suggest, however, that a revised version might prove acceptable, if you can address their concerns. If you think that you can deal satisfactorily with the criticisms on revision, I would be pleased to see a revised manuscript. We would then return it to the reviewers.

At this stage, we also ask you to ensure your manuscript complies with our formatting guidelines. Provided you are able to fully address the referees' comments, we are positive about publication of your paper (we accept over 95% of revision submissions) and therefore hope you won't mind any extra work involved in reformatting your manuscript at this point.

Please upload both a 'clean' version of your Word file, along with a highlighted version clearly showing where you have made changes in the revised manuscript. Please avoid using 'Track changes' in Word files as these are lost in PDF conversion.

I should be grateful if you would also provide a point-by-point response detailing how you have dealt with the points raised by the reviewers in the 'Response to Reviewers' box. Please attend to

© 2026. Published by The Company of Biologists under the terms of the Creative Commons Attribution License (https://creativecommons.org/licenses/by/4.0/).

all of the reviewers' comments. If you do not agree with any of their criticisms or suggestions please explain clearly why this is so.

Reviewer 1

Comments for the author

In this manuscript Konstantinidi et al examine the effect of transient mRNA transfection on HBECs and their subsequent differentiation, using transient FOXJ1 mRNA transfection via PBAE nanoparticles.

They find that PBAE + FOXJ1 transfection in submerged HBEC cultures results in ~50% of cells expressing FOXJ1, but only transiently (less than 48hours) and while this results in an increase in ciliogenesis related genes (known to be targets of FOXJ1), these increases are ~1000-10,000 fold less than the normal expression of these genes during differentiation (via ALI conditions). Nevertheless, these results confirm that it is possible to induce expression of downstream target genes via non-viral mRNA-mediated expression of a transcription factor of interest in primary HBEC culture.

The authors also explore whether differentiation occurs normally after mRNA transfection via PBAE nanoparticles, and find no differences in gene expression (ciliogenesis related genes, cell adhesion markers and differentiation markers), epithelial morphology, percentage of differentiated cells, or cilia ultrastructure between transfected and non-transfected primary HBEC cells during ALI conditions.

Overall these findings are important, as they show that mRNA-mediated transfections are a valuable method for transient overexpression of proteins of interest, which has broad therapeutic applicability. It is important for the effect of these methods on epithelial integrity, cell differentiation and cilia function to be shown. While the effect of transient FOXJ1 expression was found to be minimal, this is perhaps not surprising given the experimental set up. But some conclusions have been made based around the impact of FOXJ1 over-expression which results in a disconnect between the data shown and the narrative of the paper. Specific comments are below:

Major comments:

1. The authors highlight the main finding of the paper is the potential of mRNA-mediated FOXJ1 manipulation as a novel tool for studying airway epithelial differentiation in vitro (Page 28, line 450), and that this study details the impact of an mRNA-encoded transcription factor on primary basal HBEC differentiation (page 28 line 455). However, it is unclear currently the extent of the overexpression of FOXJ1 gained by the transfection, and therefore the impact is not able to be assessed. While the mRNA is measured after transfection, the amount of protein expressed is not quantified. Other work performs multiple IVT-mRNA transfection (e.g. 5x daily transfections PMID: 37553383) to achieve a high level of protein expression over time due to the transient nature of mRNA-mediated transfections. Here only modest increases in expression from downstream targets are seen (compared to during ALI differentiation conditions) and as such, it is perhaps not surprising that the differentiation and epithelial morphology is not affected. Therefore, it is important to assess the protein level achieved from the transfections.

* Therefore, it would be informative to measure the protein expression 12 hours after transfection in comparison to 14 days after ALI culture to be able to put into context the functional relevance of the over-expression. Also including the number of FOXJ1 positive cells 14 days after ALI to compare to the transfection will be informative. If the authors find the amount of protein expressed after transfection to be substantially lower than what is expressed during ALI, some claims need to be toned down (those focused on the impact of FOXJ1), and instead could change to focus on the fact that PBAE-mediated transfection is not detrimental for HBEC differentiation rather than focusing on the role of FOXJ1, and outlining the limitations and optimisation needed to express proteins at physiological levels. Alternatively, some discussion could be included about how low levels of FOXJ1 are not sufficient to express downstream targets to physiological levels.

© 2026. Published by The Company of Biologists under the terms of the Creative Commons Attribution License (https://creativecommons.org/licenses/by/4.0/).

* Additionally, if protein levels of FOXJ1 post transfection are lower than physiological levels during ALI, an additional discussion sentence is needed (page 26, line 423), including that transient low level FOXJ1 expression alone is insufficient to drive full ciliated cell maturation under submerged conditions, and that additional differentiation cues are required, and/or higher expression of FOXJ1.

2. The authors state that "Collectively, these findings demonstrate that mRNA-mediated FOXJ1 overexpression accelerates the activation of multiple ciliogenesis-related genes in primary HBECs prior to ALI establishment." (Page 18, line 347). Figure 2B-J contains no statistical information to understand which time points have increased expression, and the word "accelerate" implies a shift in timing of differentiation which is not reflected in the data. The wording of this claim should be changed, and instead discuss the time points that do show differences in expression.

3. The authors state that epithelial integrity has been maintained after PBAE+FOXJ1 transfection, although this is not actually tested. They use expression of adhesion genes and TEM images, and while there are no differences between transfected and transfected using these assays, a test of integrity is needed to make these claims. Transepithelial electrical resistance (TEER) of ALI differentiated cells would be a good method to measure barrier formation. Alternatively, these claims could be toned down to instead mention that the morphology of the epithelia, and expression of adhesion related genes are unaffected.

4. The authors mention that endogenous FOXJ1 expression in un-transfected cells remained lower than in transfected cells throughout the differentiation (page 18, line 326). However, Figure 2B shows very similar expression from day 7 onwards, and statistical information is required to make this claim.

5. Figure legends do not include information about the statistical tests used, or it is unclear if a statistical test was used. Please update this information for Figure 1D, F, Figure 2B-J, Figure 3 A-C, Figure 4A-D, F (including mentioning if no stars are included in the figure then the comparison was found to be not significant).

6. It is very interesting that a higher dose of mRNA does not equate to more cells expressing FOXJ1 protein. Does the level of protein expression change with increasing mRNA dose? The claims of increased cytotoxicity is also not supported. Supp Figure 4 needs statistical analysis to compare un-transfected to PBAE+FOXJ1 transfections, as these look very similar between all doses. Other papers are also able to transfect much higher amounts than used in this paper, and therefore more discussion is needed here. If possible, this work would also benefit from a comparison between comparing GFP transfections and FOXJ1 transfections at different doses if the authors believe that it the lack of a dose response is FOXJ1-specific.

Minor comments

* Fig 4F does not have a legend to indicate which colour bars represent which condition.

* Multiple primary cell donors are mentioned in figures, but information about these donors are missing from the methods. Are they all from healthy donors, what is known about their demographies, etc? If some of the information is unknown, then catalogue numbers will suffice.

* Please include the collagen concentration used for coating tissue culture flasks and inserts.

<u>Reviewer 2</u>

Comments for the author

This study investigates the delivery of IVT-mRNA encoding the transcription factor FOXJ1 to primary human airway epithelial cells and examines its effects on ciliogenesis and epithelial differentiation. The authors demonstrate efficient, transient FOXJ1 expression following nanoparticle-mediated mRNA delivery and show that this induces early expression of ciliogenesis-associated genes without disrupting epithelial integrity or long-term differentiation at air-liquid

© 2026. Published by The Company of Biologists under the terms of the Creative Commons Attribution License (https://creativecommons.org/licenses/by/4.0/).

interface (ALI). While many of the findings are expected given the established role of FOXJ1, the work offers some novelty as a technical platform for transient manipulation of gene expression in primary airway epithelial cells.

That said, the rationale for the study is not clearly articulated. IVT-mRNA delivery is indeed of therapeutic interest, and its effects on airway epithelial differentiation may not be fully characterised. However, this does not, in itself, provide a strong justification for ectopic expression of FOXJ1, a well-established master regulator of ciliogenesis. As presented, the study effectively tests whether forced FOXJ1 expression perturbs normal differentiation programs and largely finds that it does not. This is a valid and potentially useful conclusion, but it is not framed clearly in the manuscript.

Several specific points merit attention:

* In Figure 1C, naked FOXJ1 mRNA data appear to be missing (no pink symbols are visible). If naked mRNA results are uniformly zero, this should be stated explicitly.

* The Figure 1F legend should clearly state how the data were generated (qPCR vs RNA sequencing). In addition, the rationale for selecting the specific genes shown should be described.

* In submerged cultures, FOXJ1 expression peaks at >50% of cells at 12 hours but declines to ~5% by 48 hours. This strongly suggests that the approach induces only transient changes in gene expression and does not establish stable transcriptional or differentiation states. This point is important and should be more clearly acknowledged.

* The authors state that FOXJ1 mRNA accelerates ciliogenesis in ALI cultures; however, in Figure 2, FOXJ1 itself appears to be the primary gene showing statistically significant early differences. It is not clear which downstream genes robustly support the claim of accelerated ciliogenesis.

* In Figure 3, ciliated cells are present in both control and FOXJ1-transfected cultures at late ALI time points, as expected. Since these cells would have differentiated regardless of transfection, it is difficult to draw strong conclusions from these comparisons. Similarly, it would not be expected that FOXJ1 overexpression alters epithelial barrier integrity, so these negative results, while reassuring, are not particularly informative.

FOXJ1 mRNA expression does not measurably alter epithelial differentiation capacity, either by qPCR analysis of marker genes or by immunofluorescence-based cell counting. This is an important result but is currently under-emphasised.

* Are the high dose mRNA cells exposed to more PBAE? Could the apparent lower expression be due to the reagent rather than high concentrations of mRNA. It seems a very narrow window for optimal expression.

* The submerged culture experiments (Figure 5) would benefit from clearer methodological description. It is unclear whether these were proliferating cultures maintained long-term in growth-promoting medium, in which case the absence of ciliogenesis is unsurprising. Additionally, α-tubulin staining alone is insufficient to demonstrate ciliogenesis, as it can also be induced by cellular stress. Recent work (e.g. Dreyer et al., 2024, bioRxiv: https://doi.org/10.1101/2024.10.17.618953) showing ciliogenesis in submerged conditions may be relevant for discussion.

* Finally, I do not agree with the conclusion that the findings "establish a foundation for leveraging mRNA technology to manipulate pathways involved in airway epithelial differentiation." Rather, the data suggest that airway epithelial differentiation programs are remarkably robust, and that transient ectopic expression of a master regulator such as FOXJ1 produces only short-term transcriptional effects without altering long-term differentiation outcomes. This is a valid and interesting conclusion, but it should be stated more clearly and with appropriate caution.

* N numbers should be clarified in legends (including technical vs experimental replicates)

© 2026. Published by The Company of Biologists under the terms of the Creative Commons Attribution License (https://creativecommons.org/licenses/by/4.0/).

Overall, I feel the manuscript would be significantly strengthened by a clearer articulation of its purpose, a more restrained interpretation of the findings, and a more explicit discussion of the limitations of transient mRNA-mediated transcription factor overexpression.

Typo:

Line 510: "for the findings support the utility of primary human ALI" - grammatical error.

Reviewer's Responses to Questions

Experimental quality

Does each figure have the proper controls?
If 'No', please indicate reasons in Comments for Author box below.
Reviewer #1:

- Yes

Reviewer #2:

- Yes

Were the data analyzed using appropriate statistical tests?
If 'No', please indicate reasons in Comments for Author box below.
Reviewer #1:

- No

Reviewer #2:

- Yes

Reproducibility
Were experiments performed using adequate number of biological replicates?
If 'No', please indicate reasons in Comments for Author box below.
Reviewer #1:

- Yes

Reviewer #2:

- No

Does the methods section provide sufficient detail to permit reproducibility?
If 'No', please indicate reasons in Comments for Author box below.
Reviewer #1:

- No

Reviewer #2:

- Yes

Completeness
Are the manuscript's conclusions supported by the data?
If 'No', please indicate reasons in Comments for Author box below.
Reviewer #1:

- No

Reviewer #2:

- No

© 2026. Published by The Company of Biologists under the terms of the Creative Commons Attribution License (https://creativecommons.org/licenses/by/4.0/).

Scholarship
Do the authors cite and discuss the merits of data that would argue for and against their conclusion?
If 'No', please indicate reasons in Comments for Author box below.
Reviewer #1:
- No

Reviewer #2:
- Yes

Does the manuscript title & abstract accurately reflect the contents of the manuscript, without hyperbole?
If 'No', please indicate reasons in Comments for Author box below.
Reviewer #1:
- No

Reviewer #2:
- No

First revision

Author response to reviewers' comments

We would like to thank the reviewers for taking time to provide valuable feedback on our manuscript which we think has improved the revised article.

Please find below responses to comments and corresponding changes in the manuscript:

Reviewer 1: Overall these findings are important, as they show that mRNA-mediated transfections are a valuable method for transient overexpression of proteins of interest, which has broad therapeutic applicability. It is important for the effect of these methods on epithelial integrity, cell differentiation and cilia function to be shown. While the effect of transient FOXJ1 expression was found to be minimal, this is perhaps not surprising given the experimental set up. But some conclusions have been made based around the impact of FOXJ1 over-expression which results in a disconnect between the data shown and the narrative of the paper. Specific comments are below:

Major comments:

1. The authors highlight the main finding of the paper is the potential of mRNA-mediated FOXJ1 manipulation as a novel tool for studying airway epithelial differentiation in vitro (Page 28, line 450), and that this study details the impact of an mRNA-encoded transcription factor on primary basal HBEC differentiation (page 28 line 455). However, it is unclear currently the extent of the overexpression of FOXJ1 gained by the transfection, and therefore the impact is not able to be assessed. While the mRNA is measured after transfection, the amount of protein expressed is not quantified. Other work performs multiple IVT-mRNA transfection (e.g. 5x daily transfections PMID: 37553383) to achieve a high level of protein expression over time due to the transient nature of mRNA-mediated transfections. Here only modest increases in expression from downstream targets are seen (compared to during ALI differentiation conditions) and as such, it is perhaps not surprising that the differentiation and epithelial morphology is not affected. Therefore, it is important to assess the protein level achieved from the transfections.

*** Therefore, it would be informative to measure the protein expression 12 hours after**

© 2026. Published by The Company of Biologists under the terms of the Creative Commons Attribution License (https://creativecommons.org/licenses/by/4.0/).

transfection in comparison to 14 days after ALI culture to be able to put into context the functional relevance of the over-expression. Also including the number of FOXJ1 positive cells 14 days after ALI to compare to the transfection will be informative. If the authors find the amount of protein expressed after transfection to be substantially lower than what is expressed during ALI, some claims need to be toned down (those focused on the impact of FOXJ1), and instead could change to focus on the fact that PBAE-mediated transfection is not detrimental for HBEC differentiation rather than focusing on the role of FOXJ1, and outlining the limitations and optimisation needed to express proteins at physiological levels. Alternatively, some discussion could be included about how low levels of FOXJ1 are not sufficient to express downstream targets to physiological levels.

*** Additionally, if protein levels of FOXJ1 post transfection are lower than physiological levels during ALI, an additional discussion sentence is needed (page 26, line 423), including that transient low level FOXJ1 expression alone is insufficient to drive full ciliated cell maturation under submerged conditions, and that additional differentiation cues are required, and/or higher expression of FOXJ1.**

Response: We agree with the reviewer and have changed focus as they suggest to focus 'on the fact that PBAE-mediated transfection is not detrimental for HBEC differentiation' and have removed emphasis on the role of FOXJ1.

We agree that without direct FOXJ1 protein quantification we cannot benchmark the magnitude of FOXJ1 expression achieved by IVT-mRNA transfection against physiological FOXJ1 levels during ALI differentiation. In the revised manuscript we have reframed the study accordingly shifting the focus from 'FOXJ1 mRNA as a tool to study airway differentiation' to evaluating whether PBAE-mediated IVT mRNA transfection of primary basal HBECs is compatible with subsequent ALI differentiation. We have changed the title and abstract to reflect this.

To support this revised focus, we have added a new figure 1 to support PBAE mediated transfection of basal cells followed by differentiation using a GFP reporter and have removed content that implied FOXJ1 mRNA delivery itself drives differentiation outcomes as the focus of the paper has changed. We describe the FOXJ1 data as evidence of transient FOXJ1 induction with modest downstream transcript changes in primary HBECs, while emphasizing that ALI differentiation outcomes were not detectably compromised by prior mRNA transfection.

Revised abstract: Lines 9-27

Revised introduction text: Lines 44-50, 51-72, 88-93

Revised results text: Lines 281-315, 338-345, 349-351, 352-354, 365-369

Revised discussion text: Lines 418-431, 449-456, 463-465, 470-476, 494-501

2. The authors state that "Collectively, these findings demonstrate that mRNA-mediated FOXJ1 overexpression accelerates the activation of multiple ciliogenesis-related genes in primary HBECs prior to ALI establishment." (Page 18, line 347). Figure 2B-J contains no statistical information to understand which time points have increased expression, and the word "accelerate" implies a shift in timing of differentiation which is not reflected in the data. The wording of this claim should be changed, and instead discuss the time points that do show differences in expression.

Response: We agree that "acceleration" may imply a shift in the rate of differentiation rather than a change in the timing of initiation. We have revised the manuscript to use more precise terms such as "induction" and "early activation". For statistical clarity, we have updated Figure 2B-J legend to include statistical analysis (Two-way ANOVA with Dunnett's multiple comparisons test; while early induction (6–48h) of downstream genes (*RFX2*, *RFX3*, *DYNC2H1*, *IFT172*) follows an upward trend in transfected cells, the differences did not reach statistical significance ($P>0.9999$). We explicitly limit statistical statements to FOXJ1 mRNA, for which significant overexpression was detected from 6–48 h post-transfection, and describe the remaining ciliogenesis-related genes

© 2026. Published by The Company of Biologists under the terms of the Creative Commons Attribution License (https://creativecommons.org/licenses/by/4.0/).

(*RSPH9*, *DNALI1, DNAH11*, *CETN2*, *RFX2/3*, *DYNC2H1*, *IFT172*) as showing earlier detectable expression and/or trends toward earlier induction in FOXJ1 mRNA- transfected cells prior to ALI establishment, without implying statistical significance. We also clarify that following air-lift, expression profiles in transfected and non-transfected cultures converge over the differentiation period, consistent with ALI conditions driving the overall differentiation trajectory.

We have focused on activation of certain downstream genes to indicate transfection has occurred and that this does not impact differentiation of HBEC after PBAE mediated delivery. We have removed suggestions of activation of pathways that might impact ciliogenesis during submerged conditions.

Revised results text: Lines 352-354, 365-369, 370-376

Revised discussion text: Lines 470-476

Revised Figure 2 legend: Lines 543-562

3. The authors state that epithelial integrity has been maintained after PBAE+FOXJ1 transfection, although this is not actually tested. They use expression of adhesion genes and TEM images, and while there are no differences between transfected and transfected using these assays, a test of integrity is needed to make these claims. Transepithelial electrical resistance (TEER) of ALI differentiated cells would be a good method to measure barrier formation. Alternatively, these claims could be toned down to instead mention that the morphology of the epithelia, and expression of adhesion related genes are unaffected.

Response: We agree that TEER and permeability assays are the gold standard for assessing functional barrier integrity. We performed TEER measurements on reporter transfected HBECs and therefore did not repeat this in FOXJ1 studies, we have now added a new Figure 1 showing these data. For FOXJ1 we focused on assessing whether mRNA-mediated transient FOXJ1 expression caused structural disruption or changes in the molecular machinery of the cell-cell junctions. We believe the combination of TEM (visualizing the physical junctions) and qPCR (confirming the presence of mRNA for tight and adherens junctions) and TEER from reporter mRNA experiments provides evidence that the epithelial morphology remains unaffected by the PBAE-mRNA transfection.

Revised methods text: Lines 245-272

Revised results text: New Figure 1, Lines 281-315, 352-354, 382-385

Revised discussion text: Lines 478-484

4. The authors mention that endogenous FOXJ1 expression in un-transfected cells remained lower than in transfected cells throughout the differentiation (page 18, line 326). However, Figure 2B shows very similar expression from day 7 onwards, and statistical information is required to make this claim.

Response: Our intended statement was that there is higher FOXJ1 mRNA at early timepoints during submerged culture prior to airlift, when endogenous FOXJ1 would normally be low. We have clarified the Results text to more accurately reflect the data: FOXJ1 expression is significantly elevated in FOXJ1 mRNA transfected cells during the early post-transfection window (6–48 h), whereas after air-lift (from day 7 onwards) FOXJ1 expression in transfected and non-transfected cultures appears comparable. We have removed the claim of persistently lower endogenous expression throughout differentiation and restricted statistical statements to the early post-transfection comparisons for which statistically significant differences are found.

Revised results text: Lines 365-369, 463-465, 470-476

© 2026. Published by The Company of Biologists under the terms of the Creative Commons Attribution License (https://creativecommons.org/licenses/by/4.0/).

5. Figure legends do not include information about the statistical tests used, or it is unclear if a statistical test was used. Please update this information for Figure 1D, F, Figure 2B-J, Figure 3 A-C, Figure 4A-D, F (including mentioning if no stars are included in the figure then the comparison was found to be not significant).

Revised figure legends: Lines 518-611

6. It is very interesting that a higher dose of mRNA does not equate to more cells expressing FOXJ1 protein. Does the level of protein expression change with increasing mRNA dose? The claims of increased cytotoxicity is also not supported. Supp Figure 4 needs statistical analysis to compare un-transfected to PBAE+FOXJ1 transfections, as these look very similar between all doses. Other papers are also able to transfect much higher amounts than used in this paper, and therefore more discussion is needed here. If possible, this work would also benefit from a comparison between comparing GFP transfections and FOXJ1 transfections at different doses if the authors believe that it the lack of a dose response is FOXJ1-specific.

Response: We agree that the absence of a clear dose - response in the percentage of FOXJ1-positive cells is interesting. We have added the corresponding mRNA level data showing that there is a dose responsive increase in amount of mRNA present intracellularly (now in Figure 2), but this does not result in an increase in the % of cells expressing the protein, this may reflect non-linear relationship of dose to the number of cells being transfected, endosomal escape or translation, across the basal HBEC population. We have revised the discussion to reflect these points. We have added statistical analysis to Supplementary Figure 4 comparing non- transfected cells to PBAE+FOXJ1 conditions across doses (test and multiple-comparisons procedure now specified in the figure legend). Based on these analyses, we have revised the wording to reflect the actual outcome.

Direct dose comparisons across publications are difficult, given variability in delivery chemistry (polymer vs lipid vectors), mRNA design (modifications, length), dosing normalization (per well vs surface area), exposure duration, cell type/differentiation state - for example higher doses are typically delivered to differentiated ALI not basal HBECs.

Revised results text: Lines 322-337

Revised discussion text: Lines 432-438, 449-465, 487-493

Revised figure legend for supplementary figure 4: supplementary figures Word document

<u>Minor comments</u>

1. Fig 4F does not have a legend to indicate which colour bars represent which condition.

Response: Now Figure 5 - we have updated the figure with figure legend.

2. Multiple primary cell donors are mentioned in figures, but information about these donors are missing from the methods. Are they all from healthy donors, what is known about their demographies, etc? If some of the information is unknown, then catalogue numbers will suffice.

Revised text: Lines 96-97

3. Please include the collagen concentration used for coating tissue culture flasks and inserts.

Revised text: Line 100

© 2026. Published by The Company of Biologists under the terms of the Creative Commons Attribution License (https://creativecommons.org/licenses/by/4.0/).

Reviewer 2: This study investigates the delivery of IVT-mRNA encoding the transcription factor FOXJ1 to primary human airway epithelial cells and examines its effects on ciliogenesis and epithelial differentiation. The authors demonstrate efficient, transient FOXJ1 expression following nanoparticle-mediated mRNA delivery and show that this induces early expression of ciliogenesis-associated genes without disrupting epithelial integrity or long-term differentiation at air-liquid interface (ALI). While many of the findings are expected given the established role of FOXJ1, the work offers some novelty as a technical platform for transient manipulation of gene expression in primary airway epithelial cells.

That said, the rationale for the study is not clearly articulated. IVT-mRNA delivery is indeed of therapeutic interest, and its effects on airway epithelial differentiation may not be fully characterised. However, this does not, in itself, provide a strong justification for ectopic expression of FOXJ1, a well-established master regulator of ciliogenesis. As presented, the study effectively tests whether forced FOXJ1 expression perturbs normal differentiation programs and largely finds that it does not. This is a valid and potentially useful conclusion, but it is not framed clearly in the manuscript.

Several specific points merit attention:

1. In Figure 1C, naked FOXJ1 mRNA data appear to be missing (no pink symbols are visible). If naked mRNA results are uniformly zero, this should be stated explicitly.

Response: Naked FOXJ1 mRNA did not yield detectable FOXJ1-positive cells under the tested conditions, resulting in 0 across replicates. We have addressed this by explicitly stating in the Results section that naked FOXJ1 mRNA produced no detectable FOXJ1 expression (0% FOXJ1-positive cells) and changed the symbols on the figure to make this clearer.

Revised results text: Lines 322-337

2. The Figure 1F legend should clearly state how the data were generated (qPCR vs RNA sequencing). In addition, the rationale for selecting the specific genes shown should be described.

Response: Data shown here were generated by qPCR analysis of FOXJ1 downstream genes at 24 hours post-transfection. We have revised Figure 1F (now Figure 2E) legend to explicitly state the experimental method. In addition, we have included a brief rationale in the legend explaining that the genes were selected as representative FOXJ1-regulated markers spanning key functional categories of ciliogenesis, namely transcriptional regulation, ciliary structure/motility, and intraflagellar transport. A more detailed discussion of gene selection remains in the Discussion section (lines 459-463).

Revised figure 2 legend: Lines 543-563

3. In submerged cultures, FOXJ1 expression peaks at >50% of cells at 12 hours but declines to ~5% by 48 hours. This strongly suggests that the approach induces only transient changes in gene expression and does not establish stable transcriptional or differentiation states. This point is important and should be more clearly acknowledged.

Response: We agree that the decline in FOXJ1-positive cells indicates that mRNA-mediated FOXJ1 overexpression induces a transient transcriptional response under submerged culture conditions. We have revised the Discussion section to explicitly acknowledge this and stated in abstract.

Revised abstract: Lines 9-27

Revised results text: Lines 332-337, 338-345, 349-351, 370-376

Revised discussion text: Lines 418-431, 463-465, 470-476, 494-501

© 2026. Published by The Company of Biologists under the terms of the Creative Commons Attribution License (https://creativecommons.org/licenses/by/4.0/).

4. The authors state that FOXJ1 mRNA accelerates ciliogenesis in ALI cultures; however, in Figure 2, FOXJ1 itself appears to be the primary gene showing statistically significant early differences. It is not clear which downstream genes robustly support the claim of accelerated ciliogenesis.

Response: This comment is similar to reviewer 1. We agree that "acceleration" may imply a shift in the rate of differentiation rather than a change in the timing of initiation. We have revised the manuscript to use more precise terms such as "induction" and "early activation". For statistical clarity, we have updated Figure 2B-J legend to include statistical analysis (Two-way ANOVA with Dunnett's multiple comparisons test; while early induction (6–48h) of downstream genes (*RFX2*, *RFX3*, *DYNC2H1*, *IFT172*) follows an upward trend in transfected cells, the differences did not reach statistical significance (P>0.9999). We explicitly limit statistical statements to FOXJ1 mRNA, for which significant overexpression was detected from 6–48 h post-transfection, and describe the remaining ciliogenesis-related genes (*RSPH9*, *DNALI1*, *DNAH11*, *CETN2*, *RFX2/3*, *DYNC2H1*, *IFT172*) as showing earlier detectable expression and/or trends toward earlier induction in FOXJ1 mRNA-transfected cells prior to ALI establishment, without implying statistical significance. We also clarify that following air-lift, expression profiles in transfected and non-transfected cultures converge over the differentiation period, consistent with ALI conditions driving the overall differentiation trajectory.

Revised results text: Lines 352-354, 365-369, 370-376

Revised discussion text: Lines 470-476

Revised Figure 2 legend: Lines 543-562

5. In Figure 3, ciliated cells are present in both control and FOXJ1-transfected cultures at late ALI time points, as expected. Since these cells would have differentiated regardless of transfection, it is difficult to draw strong conclusions from these comparisons. Similarly, it would not be expected that FOXJ1 overexpression alters epithelial barrier integrity, so these negative results, while reassuring, are not particularly informative. FOXJ1 mRNA expression does not measurably alter epithelial differentiation capacity, either by qPCR analysis of marker genes or by immunofluorescence-based cell counting. This is an important result but is currently under-emphasised.

Response: We agree that the main important focus of the paper is that the result that transfection of primary human basal cells does not impact differentiation. We have now shifted the focus onto this outcome as suggested by all reviewers and included additional reporter transfection data in Figure 1. We agree that ciliated cells arise at late ALI time points in both FOXJ1 mRNA transfected and control cultures as part of the normal differentiation program, and therefore comparisons at these stages should not be interpreted as evidence that FOXJ1 mRNA transfection drives differentiation. We have emphasised the key finding that FOXJ1 mRNA transfection does not measurably alter epithelial differentiation capacity by marker gene expression, histology/immunofluorescence-based cell-type quantification, or TEM assessment of cilia ultrastructure. We agree that these results are important because they demonstrate that transient mRNA transfection can be applied to primary HBECs without compromising their ability to undergo mucociliary differentiation in ALI culture, which supports the utility of the approach for mechanistic studies.

Revised results text: Lines 386-387, 388-398, 399-400, 412-414

Revised discussion text: Lines 418-431, 478-484, 494-501

6. Are the high dose mRNA cells exposed to more PBAE? Could the apparent lower expression be due to the reagent rather than high concentrations of mRNA. It seems a very narrow window for optimal expression.

© 2026. Published by The Company of Biologists under the terms of the Creative Commons Attribution License (https://creativecommons.org/licenses/by/4.0/).

Response:

We have updated the text to reflect that protein expression was not measured, and so dose-response of mRNA vs protein level cannot be determined. We have also added mRNA data to show that there is a dose responsive increase in uptake of mRNA at the higher dose, but this does not increase the % of cells transfected but the amount of protein produced per cell is not known. Our primary protein-level readout was the percentage of FOXJ1-positive cells, to understand the proportion of cells transfected prior to differentiation at air-lift and we therefore do not make claims regarding dose-dependent FOXJ1 protein expression. The lack of a linear dose - response in the proportion of FOXJ1-positive cells may reflect biological and delivery heterogeneity, including variable cellular uptake and/or endosomal escape across the basal HBEC population. We have revised the discussion to reflect these points.

Revised results text: Lines 322-337, 338-345

Revised discussion text: Lines 449-456

7. The submerged culture experiments (Figure 5) would benefit from clearer methodological description. It is unclear whether these were proliferating cultures maintained long-term in growth-promoting medium, in which case the absence of ciliogenesis is unsurprising. Additionally, α-tubulin staining alone is insufficient to demonstrate ciliogenesis, as it can also be induced by cellular stress. Recent work (e.g. Dreyer et al., 2024, bioRxiv: https://doi.org/10.1101/2024.10.17.618953) showing ciliogenesis in submerged conditions may be relevant for discussion.

Due to the shift in focus of the paper being on differentiation of basal cells after transfection rather than the mechanism of FOXJ1 on ciliogenesis, we have removed Figure 5.

8. Finally, I do not agree with the conclusion that the findings "establish a foundation for leveraging mRNA technology to manipulate pathways involved in airway epithelial differentiation." Rather, the data suggest that airway epithelial differentiation programs are remarkably robust, and that transient ectopic expression of a master regulator such as FOXJ1 produces only short-term transcriptional effects without altering long-term differentiation outcomes. This is a valid and interesting conclusion, but it should be stated more clearly and with appropriate caution.

We have revised the manuscript to focus on the application of transient mRNA transfection in primary HBECs using the PBAE vector, and that this does not impact differentiation. Both reviewer 1 and 2 stated this is an interesting finding but not clearly articulated. We agree this is an important finding, particularly as these PBAE-mRNA delivery systems have been used *in vivo* in multiple pre-clinical studies. The focus has been shifted to demonstrate that PBAE mediated transient transfection does not alter normal function of basal cells.

We have revised this in sections below.

Revised abstract: Lines 9-27

Revised introduction text: Lines 44-50, 51-62, 63-72, 88-93

Revised discussion text: Lines 418-431, 478-484, 494-501

9. N numbers should be clarified in legends (including technical vs experimental replicates)

We have updated all figure legends to state the number of biological replicates and technical replicates performed for each experiment.

© 2026. Published by The Company of Biologists under the terms of the Creative Commons Attribution License (https://creativecommons.org/licenses/by/4.0/).

Revised figure legends: lines 518-611

Overall, I feel the manuscript would be significantly strengthened by a clearer articulation of its purpose, a more restrained interpretation of the findings, and a more explicit discussion of the limitations of transient mRNA-mediated transcription factor overexpression.

Response: We have made changes to change the focus of the paper, including a change in the title to reflect the main findings supported by the data.

We agree with the reviewer and have changed focus as they suggest 'on the fact that PBAE-mediated transfection is not detrimental for HBEC differentiation' rather than the role of FOXJ1. In the revised manuscript we have reframed the study accordingly, shifting the focus from 'FOXJ1 mRNA as a tool to study airway differentiation' to evaluating whether PBAE-mediated IVT mRNA transfection of primary basal HBECs is compatible with subsequent ALI differentiation. We have changed the title and abstract to reflect this.

To support this revised focus, we have added a new figure 1 to support PBAE mediated transfection of basal cells followed by differentiation using a GFP reporter and have removed content that implied FOXJ1 mRNA delivery itself drives differentiation outcomes as the focus of the paper has changed. We describe the FOXJ1 data as evidence of transient FOXJ1 induction with modest downstream transcript changes in primary HBECs, while emphasizing that ALI differentiation outcomes were not detectably compromised by prior mRNA transfection.

Typo:
Line 510: "for the findings support the utility of primary human ALI" - grammatical error.

Revised conclusion paragraph: Lines 494-501

Second decision letter

MS ID#: bio.062419R1

MS Title: Investigating the impact of PBAE-mediated FOXJ1 mRNA delivery on differentiation of primary human bronchial epithelial cells

Authors: Rafaela Konstantinidi; Mira Subramanian; Laura Yates; Clare M Lloyd; Sejal Saglani; Asha Kumari Patel

I have now reached a decision on the above manuscript.

The reviewer reports are shown at the bottom of this email.

As you will see, the reviewers gave favourable reports, but raised some critical points that will require amendments to your manuscript. I hope that you will be able to carry these out, because we would like to be able to accept your paper.

At this stage, we also ask you to ensure your manuscript complies with our formatting guidelines - please see our manuscript preparation guidelines for details. Provided you are able to fully address the referees' comments, we are positive about publication of your paper (we accept over 95% of revision submissions) and therefore hope you won't mind any extra work involved in reformatting your manuscript at this point.

Please upload both a 'clean' version of your Word file, along with a highlighted version clearly showing where you have made changes in the revised manuscript. Please avoid using 'Track changes' in Word files as these are lost in PDF conversion.

© 2026. Published by The Company of Biologists under the terms of the Creative Commons Attribution License (https://creativecommons.org/licenses/by/4.0/).

I should be grateful if you would also provide a point-by-point response detailing how you have dealt with the points raised by the reviewers in the 'Response to Reviewers' box. Please attend to all of the reviewers' comments. If you do not agree with any of their criticisms or suggestions please explain clearly why this is so.

Reviewer 1

Comments for the author

Thank you for a thorough response to my comments. They have been addressed except for these two:

1. Now that the statistical information is provided in full there is a disconnect between Fig.2E and Figure 3- in that we have significant upregulation of DNALI1 and RSPH9 24hours post infection (Fig2E) while these same genes are not significantly upregulated in Figure 3 at the same time point. Both as presented as log2 fold change relative to non-transfected controls, with n=3 donors. Can the authors explain the disconnect?

2. Figure 1 is a good addition to the manuscript, detailing epithelial differentiation and integrity upon mRNA transfection of GFP. However, only N=2 has been performed for these experiments, which has prevented statistical testing in Fig1E. An explanation of technical repeat is also needed: are these independent differentiations from the same donor, or just multiple measurements taken from the same differentiation? The former can be included in the statistical testing, the later cannot. N=3 is required from all experiments, ideally from 3 different donors, but can also be achieved from independent differentiations.

Reviewer 2

Comments for the author

The manuscript is much improved by the reframing and additional experiments during revision. I have only a couple of very minor suggestions:

* "remained insignificant" (page 15, line 375) - do the authors mean non-significant?

* The strength of "functional FOXJ1 expression" claims could still be toned down. There is now some evidence for transcriptional activity but it is indirect and the ciliary function outcomes do not change.

* on page 14, line 245 the stats are unclear. The definition of significance stars should be within the Figure legend rather than the results text and the results text should make clear which genes were significantly different.

Reviewer's Responses to Questions

Experimental quality
Are experiments performed using appropriate methods that will answer the question (or test the hypothesis or support the observations) posed by the authors? Is the right tool used for the job?
If 'No', please indicate reasons in Comments for Author box below.
Reviewer #1:
- Yes

Reviewer #2:
- Yes

© 2026. Published by The Company of Biologists under the terms of the Creative Commons Attribution License (https://creativecommons.org/licenses/by/4.0/).

Were the data analyzed using appropriate statistical tests?
If 'No', please indicate reasons in Comments for Author box below.
Reviewer #1:
- Yes

Reviewer #2:
- Yes

Reproducibility
Were experiments performed using adequate number of biological replicates?
If 'No', please indicate reasons in Comments for Author box below.
Reviewer #1:
- No

Reviewer #2:
- Yes

Is there sufficient raw data to assess the rigor of the analysis?
If 'No', please indicate reasons in Comments for Author box below.
Reviewer #1:
- No

Reviewer #2:
- Yes

Does the methods section provide sufficient detail to permit reproducibility?
If 'No', please indicate reasons in Comments for Author box below.
Reviewer #1:
- Yes

Reviewer #2:
- Yes

Completeness
Are the manuscript's conclusions supported by the data?
If 'No', please indicate reasons in Comments for Author box below.
Reviewer #1:
- Yes

Reviewer #2:
- Yes

Scholarship
Do the authors cite and discuss the merits of data that would argue for and against their conclusion?
If 'No', please indicate reasons in Comments for Author box below.
Reviewer #1:
- Yes

Reviewer #2:
- Yes

© 2026. Published by The Company of Biologists under the terms of the Creative Commons Attribution License (https://creativecommons.org/licenses/by/4.0/).

Does the manuscript title & abstract accurately reflect the contents of the manuscript, without hyperbole?
If 'No', please indicate reasons in Comments for Author box below.
Reviewer #1:

- Yes

Reviewer #2:

- Yes

Second revision

Author response to reviewers' comments

Response to reviewer comments (May 2026)
We thank the reviewers for providing additional feedback on our revised manuscript and have addressed the comments below.
Reviewer 1*: Thank you for a thorough response to my comments. They have been addressed except for these two:*

1. *Now that the statistical information is provided in full there is a disconnect between Fig.2E and Figure 3- in that we have significant upregulation of DNALI1 and RSPH9 24hours post infection (Fig2E) while these same genes are not significantly upregulated in Figure 3 at the same time point. Both as presented as log2 fold change relative to non-transfected controls, with n=3 donors. Can the authors explain the disconnect?*

Response: The disconnect may reflect the different experimental set ups. Figure 2E transfection experiments were performed in 96-well plates, whereas the Figure 3 transfection experiments were performed in 24-well transwell inserts. The two culture systems differ in surface area, cell density, media volume, epithelial architecture/polarization, and porosity. These factors might influence a variety of processes that impact both the magnitude and variability of gene-expression responses. Figure legends specify the different culture formats used for Figure 2E and Figure 3.

We have revised the manuscript text to state that fold-changes in gene expression should be interpreted within each experimental setup and cannot be compared across figures due to potential impact of the culture condition. **Lines 377-382.**

2. *Figure 1 is a good addition to the manuscript, detailing epithelial differentiation and integrity upon mRNA transfection of GFP. However, only N=2 has been performed for these experiments, which has prevented statistical testing in Fig1E. An explanation of technical repeat is also needed: are these independent differentiations from the same donor, or just multiple measurements taken from the same differentiation? The former can be included in the statistical testing, the later cannot. N=3 is required from all experiments, ideally from 3 different donors, but can also be achieved from independent differentiations.*

Response: Regarding Figure 1 experiments, we have now clarified the replicates in Figure 1 legend. For each of the two primary HBEC donors, three independent ALI culture experiments were established, giving a total of six independent ALI cultures across two donors.

For the qPCR experiment shown in Figure 1E, lysates from the three ALI cultures generated from each donor were pooled prior to RNA extraction/qPCR analysis. Therefore, Figure 1E shows two donor-level datapoints, with each point representing one pooled lysate derived from three independent ALI cultures from the same donor. As the qPCR analysis was ultimately performed on two donor-level pooled samples, statistical testing was not appropriate and was therefore not performed for Figure 1E. This has now been clarified in the **Figure 1 legend, lines 521-549.**

© 2026. Published by The Company of Biologists under the terms of the Creative Commons Attribution License (https://creativecommons.org/licenses/by/4.0/).

Reviewer 2: *The manuscript is much improved by the reframing and additional experiments during revision. I have only a couple of very minor suggestions:*

Point 1: "remained insignificant" (page 15, line 375) - do the authors mean non-significant?

Response: Yes thank you – we have changed wording. **Line 376**

Point 2: The strength of "functional FOXJ1 expression" claims could still be toned down. There is now some evidence for transcriptional activity but it is indirect and the ciliary function outcomes do not change.

Response: We have removed 'functional FOXJ1 expression' claims. **Lines 316-317, 429, 469-470**

Point 3: on page 14, line 245 the stats are unclear. The definition of significance stars should be within the Figure legend rather than the results text and the results text should make clear which genes were significantly different.

Response: We have revised the Results text to specify that only *DNALI1* and *RSPH9* were significantly increased following FOXJ1 mRNA transfection, while the remaining genes showed non-significant changes. **Lines 342-346**

Third decision letter

MS ID#: bio.062419R2

MS Title: Investigating the impact of PBAE-mediated FOXJ1 mRNA delivery on differentiation of primary human bronchial epithelial cells

Authors: Rafaela Konstantinidi; Mira Subramanian; Laura Yates; Clare M Lloyd; Sejal Saglani; Asha Kumari Patel

I am happy to tell you that your manuscript has been accepted for publication in Biology Open, pending our standard publication integrity checks. It was accepted on 17th May 2026.

© 2026. Published by The Company of Biologists under the terms of the Creative Commons Attribution License (https://creativecommons.org/licenses/by/4.0/).
